# Risperidone Mitigates Enhanced Excitatory Neuronal Function and Repetitive Behavior Caused by an ASD-Associated Mutation of SIK1

**DOI:** 10.3389/fnmol.2021.706494

**Published:** 2021-07-06

**Authors:** Moataz Badawi, Takuma Mori, Taiga Kurihara, Takahiro Yoshizawa, Katsuhiro Nohara, Emi Kouyama-Suzuki, Toru Yanagawa, Yoshinori Shirai, Katsuhiko Tabuchi

**Affiliations:** ^1^Department of Molecular and Cellular Physiology, Shinshu University School of Medicine, Matsumoto, Japan; ^2^Department of NeuroHealth Innovation, Institute for Biomedical Sciences, Interdisciplinary Cluster for Cutting Edge Research, Shinshu University, Matsumoto, Japan; ^3^Research Center for Supports to Advanced Science, Shinshu University, Matsumoto, Japan; ^4^Department of Oral and Maxillofacial Surgery, Faculty of Medicine, University of Tsukuba, Tsukuba, Japan

**Keywords:** SIK1, EIEE-30, ASD, synaptic balance, risperidone

## Abstract

Six mutations in the salt-inducible kinase 1 (SIK1)-coding gene have been identified in patients with early infantile epileptic encephalopathy (EIEE-30) accompanied by autistic symptoms. Two of the mutations are non-sense mutations that truncate the C-terminal region of SIK1. It has been shown that the C-terminal-truncated form of SIK1 protein affects the subcellular distribution of SIK1 protein, tempting to speculate the relevance to the pathophysiology of the disorders. We generated SIK1-mutant (SIK1-MT) mice recapitulating the C-terminal-truncated mutations using CRISPR/Cas9-mediated genome editing. SIK1-MT protein was distributed in the nucleus and cytoplasm, whereas the distribution of wild-type SIK1 was restricted to the nucleus. We found the disruption of excitatory and inhibitory (E/I) synaptic balance due to an increase in excitatory synaptic transmission and enhancement of neural excitability in the pyramidal neurons in layer 5 of the medial prefrontal cortex in SIK1-MT mice. We also found the increased repetitive behavior and social behavioral deficits in SIK1-MT mice. The risperidone administration attenuated the neural excitability and excitatory synaptic transmission, but the disrupted E/I synaptic balance was unchanged, because it also reduced the inhibitory synaptic transmission. Risperidone also eliminated the repetitive behavior but not social behavioral deficits. These results indicate that risperidone has a role in decreasing neuronal excitability and excitatory synapses, ameliorating repetitive behavior in the SIK1-truncated mice.

## Introduction

Salt-inducible kinases (SIKs) are a family of AMP-activated protein kinase (AMPK) consisting of SIK1, SIK2, and SIK3 (Sakamoto et al., 2018; Wein et al., 2018; Sun et al., 2020). SIK proteins are distributed all over the body, and the expression of SIK1 is shown to be induced by intrinsic stimulations, including high dietary salt intake, adrenocorticotropic hormone, neurotrophic factors, and neuronal depolarization (Wang et al., 1999; Feldman et al., 2000; Takemori et al., 2002; Finsterwald et al., 2013). SIK1 protein has an N-terminal serine/threonine kinase domain, ubiquitin-associated area (UBA), proline-glutamate-serine-threonine (PEST) domain, and C-terminal nuclear localization regulatory domain (NLD). The kinase activity of SIK1 is conferred by phosphorylation of threonine at position 182 within the serine/threonine kinase domain by liver kinase B1 (LKB1) (Lizcano et al., 2004; Jaleel et al., 2005). It has been shown that the translocation of SIK1 protein differentiates the activation of downstream signaling pathways in some organs, such as the liver and adrenal gland (Koo et al., 2005; Yoon et al., 2009). Substrates of SIK1, such as cAMP-regulated transcriptional coactivators (CRTC) and the class IIa histone deacetylases (HDAC), are distributed both inside and outside of the nucleus of the cell. Thus, the cellular localization of SIK1 protein is an important determinant of SIK1-dependent signaling pathways. The NLD of SIK1 regulates the cellular localization of SIK1 protein without altering the intrinsic kinase activity.

Six mutations in the SIK1 gene were identified in the patients with early infantile epileptic encephalopathy (EIEE-30) (Hansen et al., 2015). Two patients died before they turned 1 year old. The remaining four showed autistic symptoms, including absent language, impaired socialization, and repetitive behavior. Out of these patients with autism, two had missense mutations and the other two had nonsense mutations within NLD-coding exon, resulting in the C-terminal truncation of the SIK1 protein (Hansen et al., 2015). This report demonstrated that the C-terminal-truncated mutations seem to affect the localization of SIK1 protein. Considering the symptoms of human cases, the mutations alter the neuronal functions related to the symptoms of autism spectrum disorder (ASD).

In this study, we generated SIK1-mutant (SIK1-MT) mice lacking NLD using CRISPR/Cas9-mediated genome editing, as disease models of the human cases. We studied these mice by focusing on the behaviors and the synaptic function of pyramidal neurons in the deep layer of the medial prefrontal cortex (mPFC), where the relevance to autistic symptoms is suggested (Rinaldi et al., 2008; Yizhar et al., 2011; Brumback et al., 2018). We identified that the SIK1-MT mice showed an imbalance between excitatory and inhibitory synaptic functions by increased excitatory synaptic transmission level. We also found that the excitability was increased in the pyramidal neurons of the mPFC. The SIK1-MT mice showed an increase in repetitive behavior and deficits in social novelty preference that are compatible with the core symptoms of ASD. Risperidone has been used to treat some of the symptoms of ASD (Shea et al., 2004; Aman et al., 2005), but the pharmacological mechanisms are still unclear. In the present study, we also investigate the physiological characteristics of risperidone-treated neurons and found that risperidone rescued those synaptic abnormalities and repetitive behavior, whereas it did not change the social behavior in the SIK1-MT mice.

## Materials and Methods

### Ethics and Breeding Condition of Mice

All procedures of animal experiments were reviewed by the Committee for Animal Experiments and were finally approved by the president of Shinshu University. Mice were group-housed under environmentally controlled conditions (12:12 light:dark cycle, 22 ± 2°C, and 55 ± 10% relative humidity) with food and water *ad libitum*.

### Generation of SIK1-MT Mice and Animal Usage

We produced single guide RNA (sgRNA) targeting the SIK1 gene and mRNA of Cas9 using the MEGAscript T3 Transcription Kit (Thermo Scientific) by following the protocol of the manufacturer. For the sgRNA vector construction, annealing oligo DNAs (Oligo#1, Supplementary Table 1) were cloned into pCG-SapI (Uemura et al., 2016). sgRNA was prepared by *in vitro* transcription using PCR-amplified fragment prepared by PCR with primers (Oligo#2) with pCG-SapI-gRNA as a template. Cas9 mRNA was prepared by *in vitro* transcription using pFNLCas9A95.

Electroporation of mRNAs to the fertilized eggs from C57BL/6JmsSlc mice (Japan SLC Inc) was performed by following the TAKE method (Kaneko and Mashimo, 2015). The poring pulse was set to voltage: 40 V, pulse width: 3 ms, pulse interval: 50 ms, and the number of pulses: 4. The transfer pulse was set to voltage: 5 V, pulse width: 50 ms, pulse interval: 50 ms, and the number of pulses: ±5. After the electroporation, embryos were transferred to the oviducts of pseudo-pregnant mothers and kept until natural delivery. Fourteen pups were obtained and the InDel mutations were screened by genomic PCR and Sanger sequencing with a primer set (Oligo#3). A male mouse with 8 bp deletion that caused a frameshift in the C-terminal region of the SIK1 protein was isolated and backcrossed with C57BL/6J mice for more than six generations. All mice used in this study were littermates of C57BL/6J male and heterozygote female. The genotyping of the mice was performed by genomic PCR using primer pairs (Oligo#3), followed by the digestion of unmatched PCR products by T7 endonuclease I (New England Biolabs).

### Risperidone Treatments

To investigate the pharmacological effects of risperidone on the physiological characteristics of neurons and behavior of the SIK1-MT mice, we injected risperidone (0.05 mg/kg BW in saline, i.p.) to mice 1 h before the experiments (Burrows et al., 2015). The dose and the period were determined not to affect the activity and consciousness levels of the mice. Injected mice were returned to their home cage until further experimental procedures.

### Construction of Plasmids

Wild-type mouse brain was lysed with TRIzol RNA Isolation Reagents (Thermo Fisher), and RNA was purified by the standard phenol/chloroform extraction or with an RNA purification column, RNA Nucleospin RNA plus (Thermo Fisher). cDNA of SIK1 was obtained by standard RT-PCR using a random hexamer mixture and a primer set for SIK1 (Oligo#4, Supplementary Table 1). PCR amplicon was subcloned into pCR-Blunt TOPO vector (pCR-SIK1) and confirmed the sequence of SIK1 cDNA. pCR-SIK1 was subjected to the mutagenesis and the 8-bp deletion was introduced using a primer pair (Oligo #5) to construct pCR-SIK1mt. cDNAs of SIK1 and SIK1-MT were cloned into pCAGGS vector (pCAGGS-SIK1 and pCAGGS-SIK1mt, respectively). For the overexpression of SIK1 variants, we made cDNAs of SIK1 fused with Venus YFP (Nagai et al., 2002) at N-terminal of SIK1. Venus YFP was amplified by PCR using a set of primers (Oligo #6), and Venus cDNA amplicon was inserted into pCR-SIK1 using In-Fusion HD Cloning Kit (Takara).

### Cell Culture

HEK-293T cells (8 × 10^4^) were plated on 24-well plates 1 day before the transfection. They were transfected with 2 μg of pCAGGS-Venus-SIK1-WT, SIK1-MT, or -Q614X using polyethylenimine. Forty-eight hours after transfection, they were incubated with forskolin (10 mg/mL) and IBMX (3-isobutyl-1-methylxanthine, 18 mg/mL) or with a control vehicle (dimethyl sulfoxide). After 3 h, the cells were fixed with 4% paraformaldehyde and 4% sucrose in phosphate-buffered saline (PBS), followed by washing the cells for three times with PBS. Cells were counterstained with DAPI (4',6-diamidino-2-phenylindole). Fluorescence images were taken by a confocal laser-scanning microscope (TCS SP8; Leica Microsystems).

### Quantification of SIK1-MT mRNA Levels

To quantify the expression levels (ELs) of mRNA of the SIK1-MT, we utilized a sequencing-based quantification method (Carr et al., 2009). First, we amplified PCR products using cDNA from wild-type or SIK1-MT mice using a primer set (Oligo #7). PCR products were also prepared using pCAGGS-SIK1 plasmid or a mixture of pCAGGS-SIK1 and pCAGGS-SIK1mt at 1:1 or 1:0.5 ratio as templates. PCR amplicons from plasmids were used as standards for quantitative estimation. The PCR amplicons were subjected to Sanger sequencing, and the fluorescent data from the sequences around the sgRNA target site were analyzed by the EditR program (Kluesner et al., 2018). The ratio between wild-type and mutant sequences was calculated in each nucleotide and converted into a vector. The EL was calculated by the following formula:

(1) $$\begin{array}{l} EL\;=\;∥\vec{s}\;-\;\vec{s_{wt}}∥/∥\vec{s_{wt}}\;-\;\vec{s_{mt}}∥ \end{array}$$

Here, $\vec{s},\;\vec{s_{wt}},\;and\;\vec{s_{mt}}$ represent the vectors obtained by the calculation using data from samples from brain cDNA, pCAGGS-SIK1, and a mixture pCAGGS-SIK1 and pCAGGS-SIK1mt at 1:1 ratio (100% MT). We also calculated the EL using the PCR product from another mixture of pCAGGS-SIK1 and pCAGGS-SIK1mt at 1:0.5 ratio (50% MT). Most of the EL distributed zero to one, and a value close to one indicates that the EL of the SIK1mt is similar to that of the wild-type SIK1.

### Histology

Histological analysis was followed by previous procedures (Mori and Morimoto, 2014; Mori et al., 2019). Briefly, under deep anesthesia, 2-month-old male wild-type and SIK1-MT mice were perfused transcardially with ice-cold PBS (pH 7.4), followed by 4% paraformaldehyde in PBS. Fifty-micrometer-thick coronal sections were prepared with a sliding microtome (REM-700, Yamato Kohki Industrial). The sections were washed with PBS; blocked with PBS containing 1% bovine serum albumin, 0.1% Triton-X-100, and 10% of normal donkey serum; and incubated with mouse anti-parvalbumin (1:2000, Sigma), rat anti-somatostatin (1:200, Merck Millipore), or rabbit anti-Satb2 antibody (1:200, Abcam). After overnight incubation with primary antibodies, the brain sections were washed with PBS containing 0.1% Triton-X-100 and incubated with Alexa 488-conjugated donkey antibody against rabbit IgG or Cy2-conjugated donkey antibody against mouse or rat IgG (Jackson Immunoresearch), respectively, for 2–3 h at room temperature. After further washing with PBS, brain sections were mounted on a sliding glass, counterstained with DAPI, and coverslipped. Fluorescence images were taken with an all-in-one fluorescent microscope (BZ-X710, Keyence) and a confocal laser-scanning microscope (TCS SP8; Leica Microsystems).

### Electrophysiology

Patch-clamp recordings in acute brain slices were done as described previously (Mori et al., 2019). Briefly, postnatal 14- to 19-day-old male and female mice brains were removed and placed immediately in ice-cold slicing artificial corticospinal fluid (ACSF, in mM: 85 NaCl, 75 sucrose, 2.5 KCl, 1.25 NaH_2_PO_4_, 24 NaHCO_3_, 25 glucose, 0.5 CaCl_2_, and 4 MgCl_2_) saturated with 95% O_2_/5% CO_2_ for 2 min. Three hundred fifty-micrometer-thick coronal sections were transferred to a recovery chamber filled with recording ACSF (in mM: 126 NaCl, 2.5 KCl, 1.25 NaH_2_PO_4_, 26 NaHCO_3_, 10 glucose, 2 CaCl_2_, and 2 MgCl_2_), followed by incubation at 32°C for 30 min and then at room temperature for 30 min. In current-clamp experiments, pyramidal neurons were patched with glass pipettes (4–8 M ohm) filled with a potassium-based intracellular solution (ICS, in mM: 130 K gluconate, 6 KCl, 10 HEPES, 1 EGTA, 2.5 MgCl_2_, 2 magnesium ATP, 0.5 sodium GTP, 10 phosphocreatine sodium, 290 mOsm) under microscopy (BX50WI, Olympus). Resting membrane potential was measured immediately after establishing whole-cell recording. Hyperpolarizing and depolarizing step pulses (700 ms) were applied to characterize neuronal firing property. Membrane potential at which the temporal rate of the potential reached 10 mV/ms was defined as the action potential threshold. Postsynaptic responses were measured in a voltage-clamp mode using cesium-based ICS (in mM: 130 CsOH, 130 gluconic acid, 6 CsCl, 10 HEPES, 1 EGTA, 2.5 MgCl_2_, 2 magnesium ATP, 0.5 sodium GTP, 10 phosphocreatine sodium, 290 mOsm). Miniature postsynaptic currents were recorded in the presence of 1 mM tetrodotoxin (Abcam). To record miniature excitatory postsynaptic currents (mEPSCs), the cell membrane potential was held at −60 mV for 3 min recording sessions and then cell membrane potential was shifted to 0 mV to record mIPSCs from the same cell, for 3 min as well. Evoked postsynaptic currents were triggered with 0.1 ms current injections by a nichrome-wire electrode placed at position 100–150 μm from the soma of neurons recorded. For recording evoked AMPA and NMDA-EPSC, 100 μM picrotoxin was added in bath solution with the holding potentials at −70 and 40 mV. We used 5 mM of QX-314 in the pipette solution to block sodium channel-mediated currents. To calculate the NMDA-to-AMPA ratio, the amplitude of NMDA current at 50 ms after the onset was divided by the peak amplitude of AMPA current. Paired-pulse ratio (PPR) was done holding the cell at −70 mV for excitatory PPR (ePPR) in the presence of 100 μM picrotoxin. For inhibitory PPR (iPPR), the cells were held at 0 mV in the presence of 20 μM DNQX. All PPR experiments were done with (30, 50, 100, 200 ms) stimulation intervals. Access resistance was monitored throughout the recording, and cells with access resistance over 25 MΩ were rejected. All data were acquired at 10 kHz with an EPC10 double amplifier (HEKA) operated by Patch Master software (HEKA). Data analysis was performed with the Mini Analysis Program (Synaptosoft) and custom-made programs of Igor Pro (WaveMetrics).

### Behavioral Studies

Two- to three-month-old male heterozygote SIK1-MT and the littermate wild-type mice were used in all behavioral experiments except for the experiment to record the ultrasonic vocalization (USV). Stranger mice were C57BL/6J male mice from pure C57BL/6J parents. All experiments and analyses were performed blindly to the genotype and pharmacological treatment.

#### Open Field Tests

Each mouse was placed in the corner of the open field apparatus (50 × 50 × 40 cm). The apparatus was surrounded by a sound-attenuating white chest and was illuminated at ~100 lux. Subject behaviors were recorded from the above of the apparatus using a CCD camera (WAT-902B; Watec, Yamagata, Japan). Analog images were converted to digital images (720 × 480 pixels) using Monster HD264 (SKNET, Yokohama, Japan). The video frame rate was 30 frames per s (fps). The test lasted 30 min. We measured the travel distance, time spent in the center area (25 × 25 cm), vertical activity (rearing and leaning), and grooming. Part of the behavioral parameters, such as the travel distance and time spent in the center area, were analyzed using idTracker (Perez-Escudero et al., 2014) and custom-made programs run on MATLAB. Other parameters were analyzed by a trained observer.

#### Elevated Plus-Maze Test

The elevated plus-maze consisted of two open arms (25 × 5 cm) and two closed arms of the same size with 15-cm-high walls made of transparent plastic. The maze was arranged in a manner such that arms of the same type were opposite to each other, connected by a central area (5 × 5 cm), and the entire maze was elevated to a height of 50 cm above the floor. To keep the mice from falling over, the open arms were surrounded by a 3-mm-high edge wall. The maze was illuminated at ~100 lux. The animals were placed individually in the center of the maze, facing a closed arm. Mouse behaviors were recorded during a 5-min test period using a web camera (HD Webcam C615; Logicool, Tokyo, Japan). The video images (640 × 480 pixels) were recorded at 30 fps and analyzed using idTracker. The number of entries into the open arms was analyzed.

#### Marble Burying Test

Each mouse was placed into an arena (25 × 25 × 31 cm) filled with 5-cm-deep wood chip bedding (CLEA Japan, Tokyo, Japan) and was habituated to the test arena and bedding for 10 min under the illumination at 100 lux. After the habituation period, mice were returned to the transfer cage, and 16 small blue glass marbles (12.5 mm diameter) were placed evenly spaced in four rows of four. The subject mouse was again placed into the arena containing the 16 marbles. After 10 min, the subject was removed from the arena, and the number of buried marbles (defined as at least two-thirds covered) was counted.

#### Sociability and Social Novelty Tests

The apparatus was a rectangular, three-chambered box in a sound attenuation box with the illumination at 100 lux. The chamber was 20 × 40 × 25 cm in size, and the dividing walls were made from transparent Plexiglas, with small openings (5 × 3 cm) allowing access into each chamber. The mouse was placed in the central chamber and was allowed to explore the whole chamber for 10 min (the doorways into the two side chambers were opened). The two side chambers contained an inverted empty small black wire cup. A clear glass cylinder was placed on top of the inverted cup to prevent lifting or climbing on top. Following the habituation period, mice were placed back into the central chamber, and the doorways into the two side chambers were closed. In the sociability test, an unfamiliar male mouse (stranger 1, S1) that had no prior contact with the subject mouse was placed in either of the two cups, and then the doorways were unblocked. The location of S1 in the left- or right-sided chambers was systematically alternated between trials. The subject behaviors were recorded for 10 min using a CCD camera. After the sociability test, the subject mouse was again confined in the central chamber. In the social novelty preference test, a second unfamiliar male mouse (stranger 2, S2) was enclosed in the cup that had been empty (E) during the sociability test, and the doorways were again unblocked. The stranger mice were at least 2 weeks younger than the subject mice and had previously been habituated to placement in the small wire cup. In both tests, the amount of time that the subject head was within a 2 cm distance of the wire cup was measured as “time spent around the cup.”

#### Recording Ultrasonic Vocalization

Ultrasonic vocalization was recorded using a USB microphone (Ultramic 250, Dodotronic) with recording software (SEA, Sound emission analyzer, The Centro Interdisciplinare di Bioacustica e Ricerche Ambientali). P5–P14 mouse was separated from the mother and placed in a recording styrofoam box containing beddings. The number of USV in 10 min was counted by an observer who has no information on the genotype of the subjects.

### Sample Size and Statistical Analysis

Sample sizes were determined based on established practice and our previous experience in respective assays. The number of independent samples (e.g., neurons) is indicated on the graphs and the numbers of animals is indicated in the figure legends. All values represent the average of independent experiments ± SEM. The variance among the analyzed samples was similar. Statistical significance was determined by Student's *t*-test (for two groups) or a one-way ANOVA followed by Bonferroni's *post-hoc* test (for multiple groups). Statistical analysis was performed by custom-written R scripts, MATLAB (Mathworks), or Prism 6.0 (Graphpad Software Inc.). Statistical significance is indicated by asterisks (^*^p < 0.05, ^**^p < 0.01, ^***^p < 0.001). All data are expressed as means ± SEM. All the values are described in Supplementary Table 2.

## Results

### Generation of SIK1-MT Mice Using CRISPR/Cas9-Mediated Genome Editing

**Figure 1:**
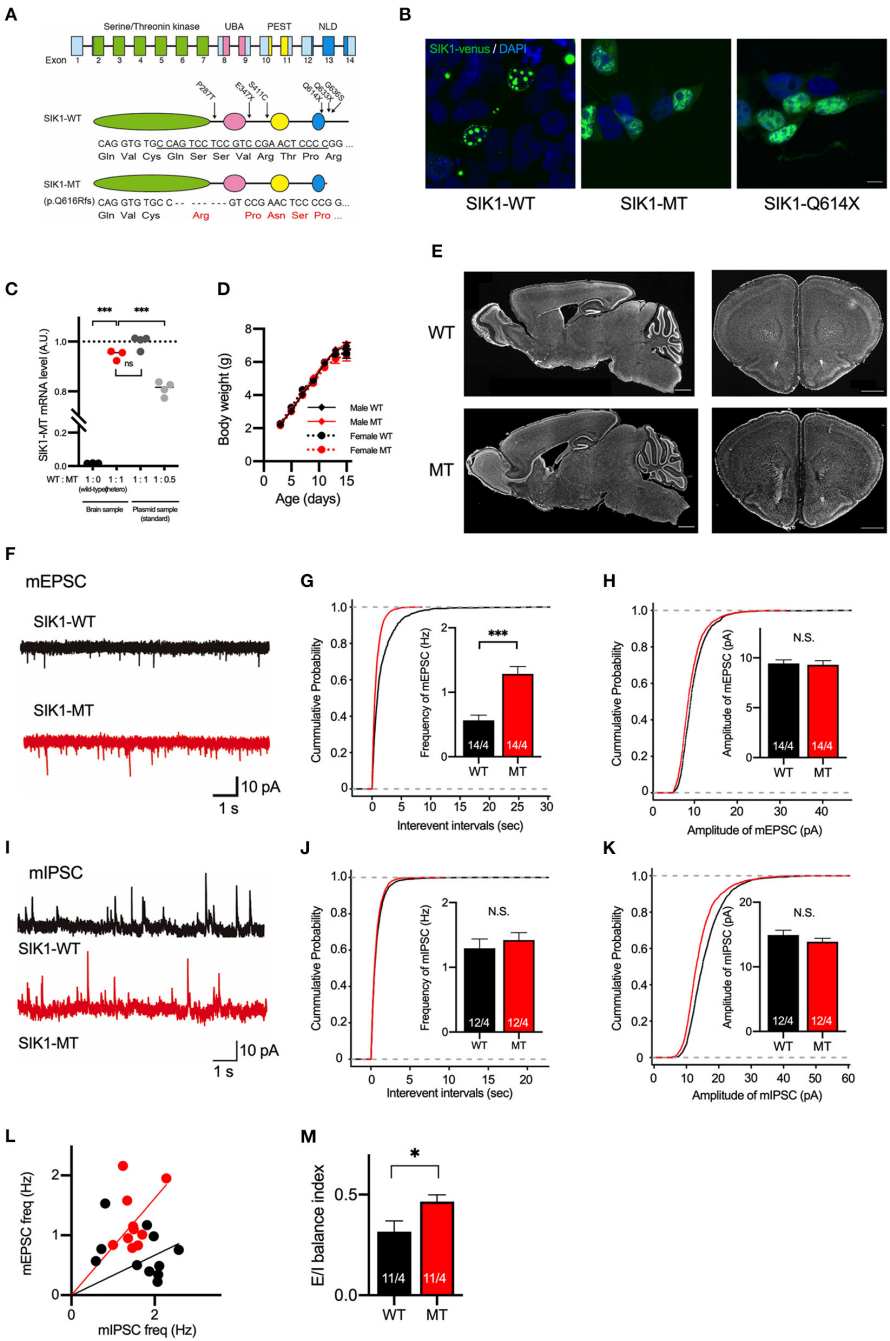
Generation and basic characterization of the SIK1 MT mice. **(A)** Genomic organization of SIK1 gene (top), domain structure of wild-type (middle), and mutant SIK1 (bottom) protein. The locations of the mutations identified in human patients are indicated on the domain structure of SIK1-WT. Nucleotide and amino acid sequences of target regions are shown. The target sequence of single guide RNA (sgRNA) is underlined. Converted amino acids after the mutated site are indicated in red. The SIK1 protein consists of four functional domains, including serine/threonine kinase, ubiquitin-associated domain (UBA), proline-glutamate-serine-threonine (PEST), and C-terminal nuclear localization regulatory domain (NLD). Fourteen exons exist in the mouse SIK1 gene. sgRNA was designed to target the coding region in exon 13 of SIK1. **(B)** Subcellular localization of Venus-tagged SIK1 mutants in HEK293T cells. Wild-type SIK1 was restricted to the nucleus in a punctate pattern, whereas SIK1-MT and pathological nonsense mutant of SIK1-Q614X were distributed both in the nucleus and in the cytoplasm. Scale bar indicates 15 μm. **(C)** The ratios between wild-type and mutant mRNA were analyzed in a wild-type, heterozygote SIK1-MT, and plasmid DNA mixture containing SIK1-MT at different ratios (WT:MT = 1:1 or 1:0.5), which are used as standards. Mutant SIK1 transcripts were detected at a similar level with plasmid DNA containing 50% of SIK1-MT. **(D)** Body weights of male wild-type (Male WT), male mutant (Male MT), female wild-type (Female WT), and female mutant (Female MT) mice are shown in the graph. Body weights are unchanged in the SIK1-MT mice. **(E)** The brain structure is unchanged in the SIK1-MT mice. Sagittal (left) and coronal (right) brain sections were stained with DAPI. Scale bars indicate 1 mm. **(F)** Representative traces of miniature excitatory postsynaptic currents (mEPSC) recorded from the layer 5 pyramidal neurons in the medial prefrontal cortex (mPFC) of wild-type and SIK1-MT mice. **(G)** Cumulative distribution of inter-event intervals of and the mean values of the frequency of mEPSCs were shown in the graph. The frequency of mEPSCs was increased in the SIK1-MT mice. **(H)** Cumulative distribution and the mean values of the amplitude of mEPSCs were shown in the graph. The amplitude of mEPSCs was unchanged in the SIK1-MT mice. **(I)** Representative traces of mIPSC recorded from the layer 5 pyramidal neurons in mPFC of SIK1-WT and SIK1-MT mice were shown. **(J)** Cumulative distribution of the inter-event intervals of mEPSC and the mean values of the frequency of mIPSCs were shown in the graph. The frequency of mIPSCs was unchanged in SIK1-MT mice. **(K)** Cumulative distributions and mean values of the amplitude of mIPSCs were shown in the graph. The amplitude of mIPSCs was unchanged in SIK1-MT mice. **(L)** The scatter plot shows the relationship between the frequencies of mIPSC (x-axis) and mEPSC (y-axis). Each dot represents a single neuron from WT (black) or MT (red). **(M)** Excitatory and inhibitory (E/I) synaptic balance index of WT and SIK1-MT mice is shown. E/I balance in SIK1-MT is shifted to excitatory dominance. The numbers of neurons and mice used in each analysis are shown on the bar (neurons/mice) in the graphs. Statistical analysis was made by a one-way ANOVA followed by Bonferroni's *post-hoc* test (for the EL of SIK1 gene expression) or Student's *t*-test (for the mean of the synaptic parameters). Statistical significance was indicated by asterisks (*p < 0.05 and ***p < 0.001).

Truncations of the SIK1 gene in the two patients with EIEE-30 occur within an NLD in the C-terminal region (Hansen et al., 2015). To generate mutant mice recapitulating the human cases as disease models, we employed CRISPR/Cas9-mediated genome editing technology. We designed an sgRNA targeting a location near one of the mutation sites found in the patients. We electroporated the sgRNA and Cas9 mRNA in fertilized eggs at the one-cell stage and transferred them to the oviducts of pseudo-pregnant mothers. We obtained 14 offsprings: Three had InDel mutations and 11 had no mutation. One of the mutant mice had a deletion of eight nucleotides, resulting in the production of the C-terminal-truncated form of SIK1 protein, resembling that in human patients (SIK1-MT, Figure 1A). SIK1 mutations found in patients were hemizygotic. We crossed these mutant mice with C57Bl/6J mice for more than six generations and used heterozygote mutant mice for all the experiments as disease models.

The previous report by Hansen et al. demonstrated that wild-type and truncated mutant SIK1 proteins were differently distributed in the cells (Hansen et al., 2015; Pröschel et al., 2017). To examine the subcellular distribution of the SIK1-MT, we transfected HEK293T cells with plasmids expressing wild-type (SIK1-WT) or mutant SIK1 proteins fused with a yellow fluorescent protein (Venus). As expected, SIK1-WT was restricted to the nucleus of HEK293T cells in a punctate fashion (Figure 1B, Supplementary Figure 1). In contrast, SIK1-MT and SIK1-Q614X, a non-sense mutation found in the human patient, were diffusely distributed in the nucleus and cytoplasm (Figure 1B). It is known that SIK1 protein is translocated from the nucleus to the cytoplasm when phosphorylated at S577 by PKA (Katoh et al., 2002). To examine whether the cellular distribution of S577-phosphorylated SIK1 mutants was altered, we incubated these transfected HEK293T cells with an adenylate cyclase activator, forskolin, and a phosphodiesterase inhibitor, IBMX, to activate PKA. SIK1-WT was translocated from the nucleus to the cytosol by PKA activation (Supplementary Figure 1), mimicking the localization of SIK1-MT and SIK1-Q614X. The activation of PKA did not alter the localization of SIK1-MT and SIK1-Q614X. These results suggest that SIK1-MT replicates the characteristics of the C-terminal-truncated mutants found in EIEE30 patients and the S577-phosphorylated form of SIK1-WT.

In the heterozygote SIK1-MT mice, the EL of SIK1-MT mRNA was comparable to that of SIK1-WT mRNA (Figure 1C). SIK1-MT mice grow normally without showing early lethality or epileptic seizures (Figure 1D). No gross morphological abnormality was observed in the SIK1-MT brain (Figure 1E). The distribution of neuronal markers, including parvalbumin, somatostatin, and Satb2, was also unaltered in the SIK1-MT brain (Supplementary Figure 2).

### Excitatory Synaptic Function Is Increased in Pyramidal Neurons in Layer 5 of the Medial Prefrontal Cortex in SIK1-MT Mice

Patients with SIK1 truncation have been reported to exhibit autistic behavioral phenotypes, such as social deficits and repetitive behavior (Hansen et al., 2015). Recent studies indicate that the deep layer of the mPFC is responsible for these behavioral abnormalities in both humans and rodents (Baron-Cohen et al., 1994; Castelli et al., 2002; Yizhar et al., 2011). Thus, we studied the neuronal function in layer 5 of the mPFC in SIK1-MT mice. First, we measured mEPSCs by whole-cell recording in a voltage-clamp mode (Figures 1F–H). We observed that the frequency, but not amplitude, of mEPSCs was significantly increased in SIK1-MT mice (Figures 1F–H). The rise and decay times of mEPSCs were not changed in SIK1-MT mice (Supplementary Figure 3). We did not observe changes in frequency, amplitude, rise time, and decay time of miniature inhibitory postsynaptic currents (mIPSCs) in SIK1-MT mice (Figures 1I–K, Supplementary Figure 3), resulting in the excitatory shift of synaptic function (Figures 1L,M). The increase in the frequency of mEPSCs is considered to be due to either the increase of the excitatory synapse number or release probability of synaptic vesicles. To discriminate these possibilities, we measured the PPR. We found that the AMPA receptor-mediated PPR, as well as GABA receptor-mediated, was unchanged in all stimulation intervals analyzed (Supplementary Figure 4), indicating that the number of the functional excitatory synapse was increased in SIK1-MT mice. We also examined the composition of postsynaptic glutamatergic receptors as a ratio of the action potential-dependent NMDA and AMPA receptor-mediated postsynaptic currents, but no change was detected in this ratio (Supplementary Figure 5).

### Excitability of Pyramidal Neurons in Layer 5 of the mPFC Is Increased in the SIK1-MT Mice

**Figure 2:**
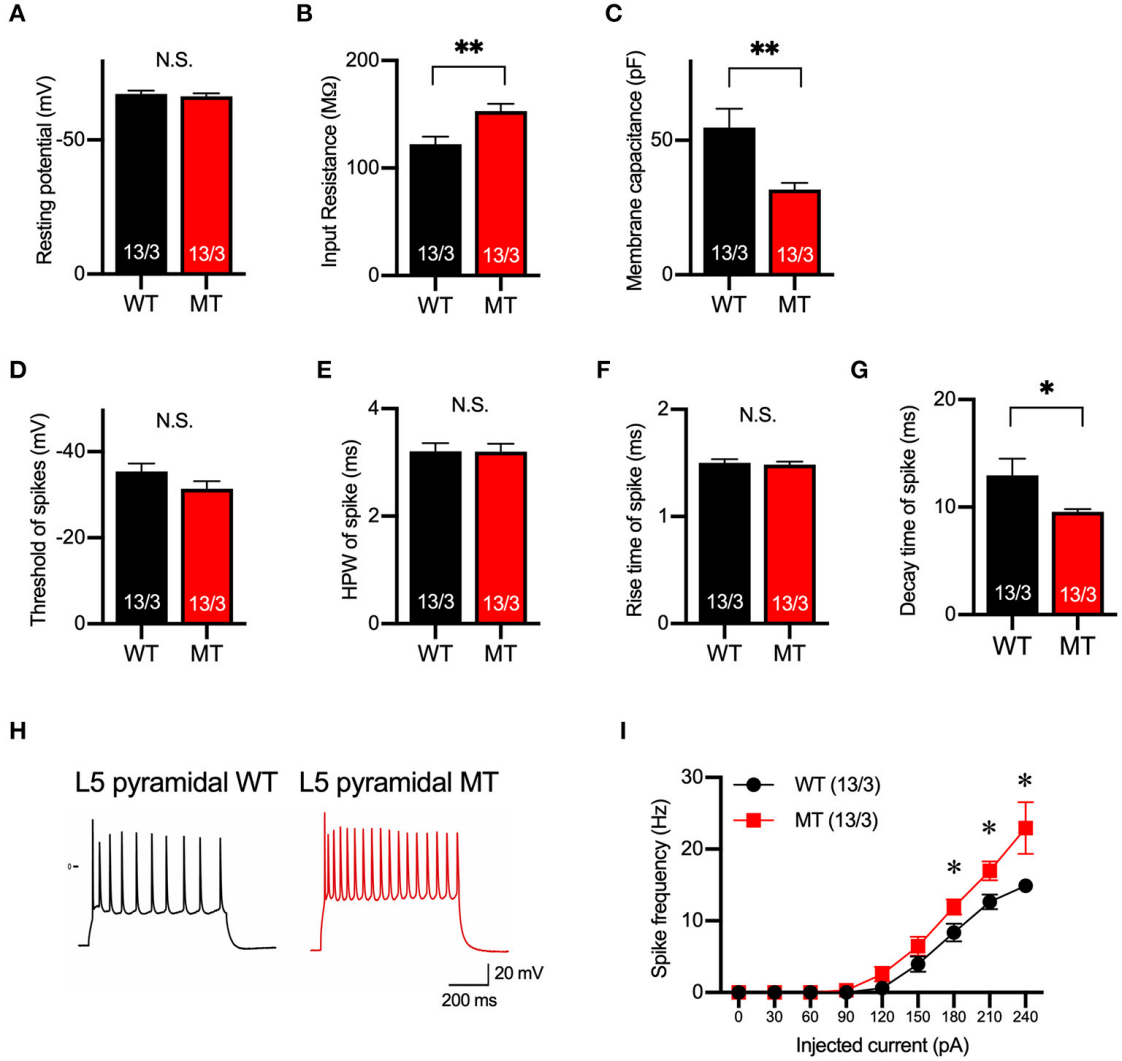
Membrane and firing properties were changed in pyramidal neurons in layer 5 of the mPFC. **(A–C)** Resting membrane potential **(A)**, input resistance **(B)**, membrane capacitance **(C)** of pyramidal neurons in layer 5 of mPFC of SIK1-WT or -MT mice. **(D–G)** threshold **(D)**, half-peak width **(E)**, rise time **(F)**, and decay time **(G)** of the action potential of the pyramidal neurons in layer 5 of the mPFC. **(H)** Representative traces of induced action potentials responded to 240 pA injected currents. **(I)** Graph for the relationship between spike frequency and injected current. Input resistance and spike frequency of action potential were increased, and membrane capacitance and decay time of action potential were decreased in SIK1-MT mice. The numbers of neurons and mice used in each analysis are shown on the bar (neurons/mice) in the graphs. Statistical analysis was made by Student's *t*-test. Statistical significance was indicated by asterisks (*p < 0.05 and **p < 0.01).

Next, we examined the membrane property and excitability of layer 5 pyramidal neurons in the mPFC of the SIK1-WT and SIK1-MT mice using whole-cell recording in a current-clamp mode. The resting potential was not changed, but input resistance was increased and membrane capacitance was decreased in the SIK1-MT mice compared with the wild-type control mice (Figures 2A–C). The threshold, half-peak width, and rise time of action potential were unchanged (Figures 2D–F), but the decay time was decreased and the spike frequency was increased in SIK1-MT mice compared with the wild-type control mice (Figures 2G–I).

### Repetitive Behavior and Social Novelty Preference Are Distorted in SIK1-MT Mice

**Figure 3:**
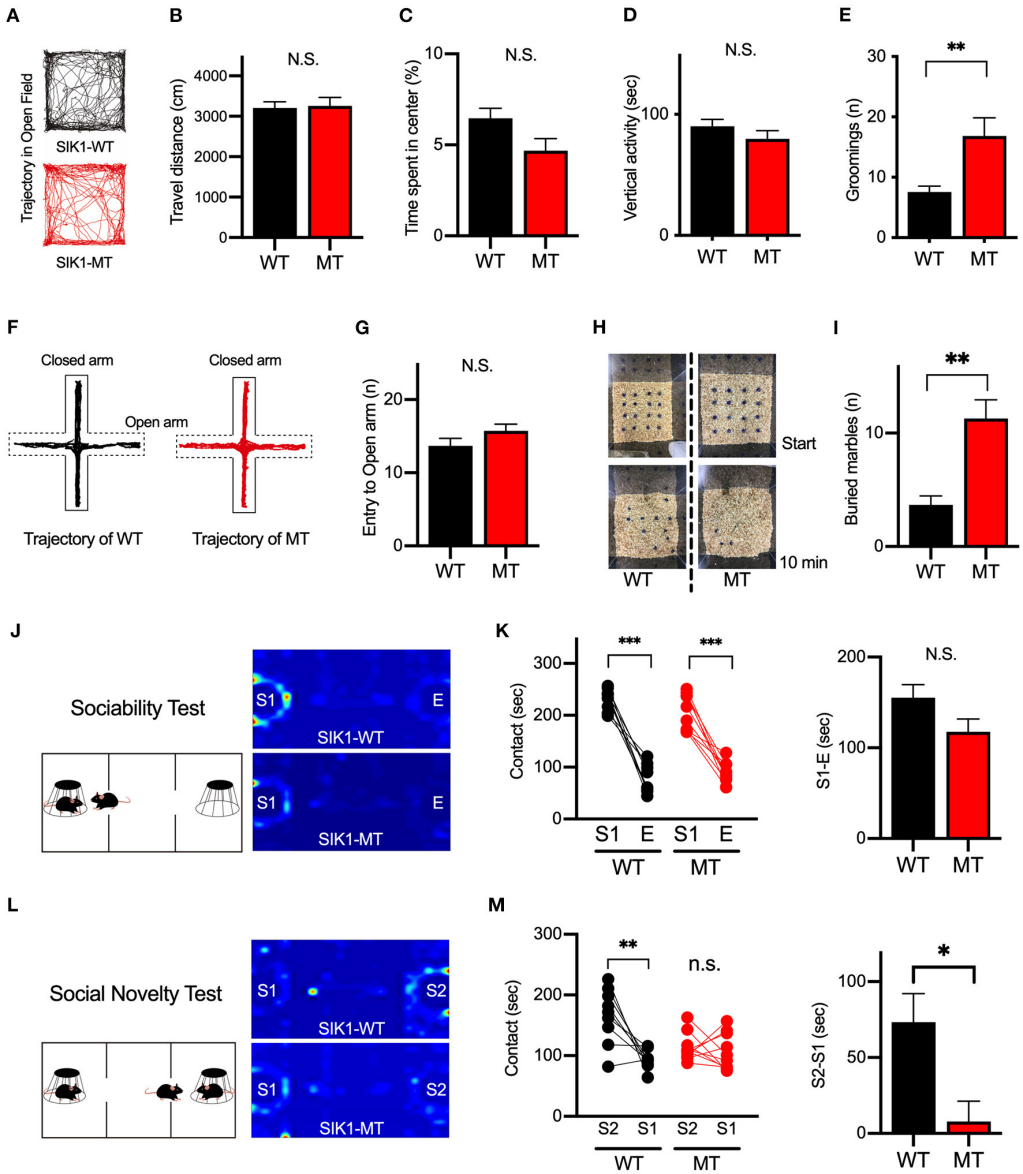
Repetitive and social behaviors were altered in SIK1-MT mice. **(A)** Trajectories of movement of SIK1-WT and -MT mice in the open field arena. **(B)** Total travel distance of SIK1-WT (*n* = 9) and -MT (*n* = 7) mice. **(C)** The percentage of the time spent in the center of the arena. **(D)** The total time of vertical activities in the open field. **(E)** The number of grooming during the observation period. The number of grooming was increased in SIK1-MT mice. **(F)** Moving trajectories of mice on the elevated plus-maze. **(G)** The number of entries into open arms of the elevated plus-maze. The number of the entry was unchanged in SIK1-MT mice. **(H)** The top views of the marble burying test field. Sixteen marbles were placed at the start of the test (top). Buried marbles were counted after 10–min of the test (bottom). **(I)** The numbers of marbles buried under the woodchip after the test. SIK1-MT mice buried more marbles in this test. **(J)** The sociability test evaluating the sociability by the time spent with the empty cage (E) vs. the cage with a stranger mouse (S1). Heatmaps represent the stay time of the test mouse. **(K)** Both WT and SIK1-MT mice interacted more with S1 than E and the preference to E vs. S1 was similar between these mice (graph). **(L)** Social novelty test evaluating social memory by the time spent with the stranger mouse (S1) versus novel stranger mouse (S2). Heatmaps represent the stay time of the test mouse. **(M)** WT mice interacted more with S2 than with S1, whereas the preference to S2 was significantly decreased in SIK1-MT mice (graph). Statistical analysis was made by Student's *t*-test. Statistical significance was indicated by asterisks (*p < 0.05, **p < 0.01, and ***p < 0.001).

To evaluate the effect of the mutation on the human cases, we examined the behaviors of the SIK1-MT mice in relevance to autistic symptoms. We first observed the general movement in the open field arena. The travel distance was unchanged, indicating that the locomotor activity was normal in the SIK1-MT mice. The time spent in the center and the vertical activity that indicates the anxiety level were also unchanged in the SIK1-MT mice (Figures 3A–D), but the SIK1-MT mice showed an increased level of grooming (Figure 3E). There was no change in movement in the elevated plus maze, which also suggests that the anxiety level was unchanged in SIK1-MT mice (Figures 3F,G). Therefore, overgrooming is suggested to reflect increased repetitive behavior. We further examined the repetitive behavior using the marble burying test (Gyertyan, 1995; Thomas et al., 2009; Hayashi et al., 2010). The number of marbles buried under the woodchip was higher in the SIK1-MT mice compared with the wild-type mice (Figures 3H,I), suggesting that the repetitive behavior was increased in SIK1-MT mice, which is consistent with the overgrooming behavior observed in the open field test.

**Figure 4:**
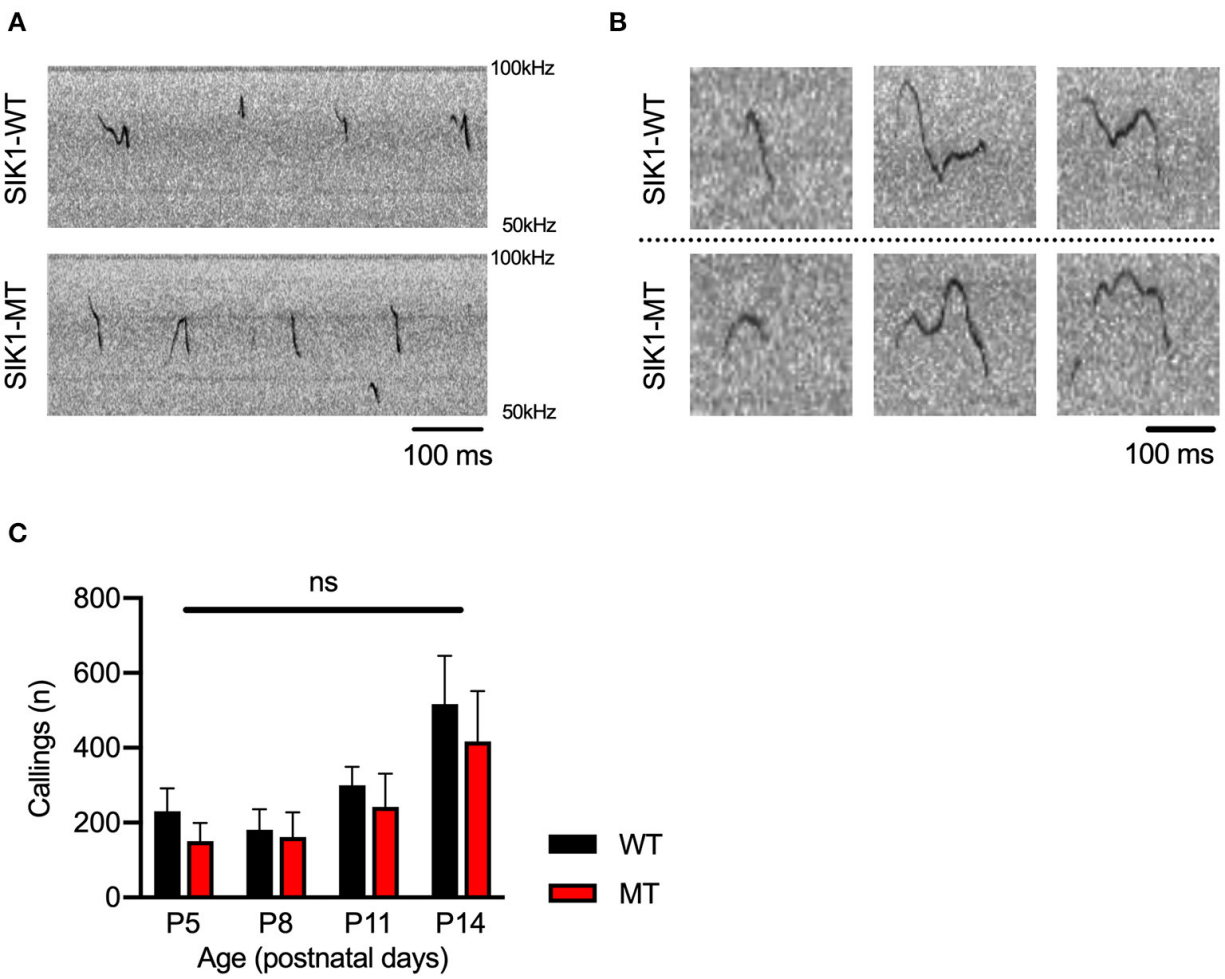
Ultrasonic vocalization was not altered between SIK1-WT and SIK1-MT mice. **(A)** Representative sonograms recorded from isolated mice (P14) were shown. **(B)** Both wild-type and mutant mice made a variety of vocalizations during the isolation from their mom. **(C)** The number of calls was not altered during the developmental stages we examined (no statistical significance was detected by *t*-test). Eight SIK1-WT and seven SIK1-MT mice were analyzed. Statistical analysis was made by Student's *t*-test.

Social deficits and language problems are the common symptoms of autism and are also reported in human cases with SIK1 mutation (Association, 2013; Hansen et al., 2015). We examined social behaviors using the three-chamber test. In the first round test, both SIK1-MT and wild-type mice showed higher interaction with the stranger mouse (S1) compared to the empty cage (E) at similar levels (Figures 3J,K). In the second round test, we placed a new stranger mouse (S2) in the empty cage and compared the interaction between S2 and the previously exposed stranger mouse in the first round test (S1) to examine the level of social novelty preference. While the interaction with S2 was higher than that with S1 in the wild-type mice, no significant difference in the interaction between S1 and S2 was observed in SIK1-MT mice (Figures 3L,M), suggesting that the social novelty preference was impaired in the SIK1-MT mice. To evaluate the vocal communication in SIK1-MT mice, we examined the USV of P5–P14 mice after separation from the mother (Liu et al., 2013). The number of the calls of USV was unaltered in SIK1-MT compared with SIK1-WT mice (Figure 4).

### Risperidone Restores the Elevated Level of Excitatory Synaptic Transmission and Excitability of Pyramidal Neurons in Layer 5 of mPFC

**Figure 5:**
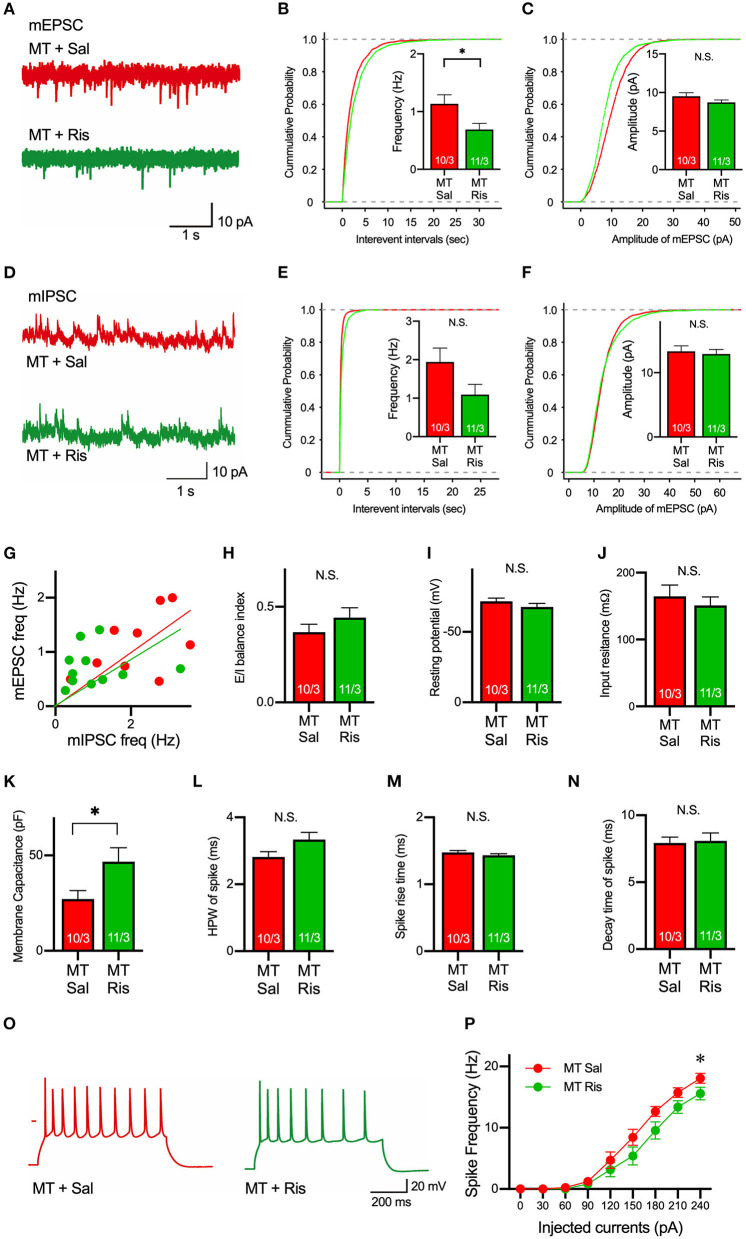
Risperidone reduces frequencies of mEPSCs and action potentials in the SIK1-MT mice. **(A)** Representative traces of mEPSC recorded from pyramidal neurons in layer 5 of the mPFC in saline (Sal)- or risperidone (Ris)-injected SIK1-MT mice. **(B)** Cumulative distribution of inter-event intervals and the mean values of the frequency of mEPSCs were shown in the graph. The frequency of mEPSCs was decreased in risperidone-injected SIK1-MT mice. **(C)**, Cumulative distribution and the mean values of the amplitude of mEPSCs were shown in the graph. The amplitude of mEPSCs was unchanged between saline- and risperidone-injected SIK1-MT mice. **(D)** Representative traces of mIPSC recorded from the layer 5 pyramidal neurons in mPFC of saline (Sal)- and risperidone (Ris)-injected SIK1-MT mice were shown. **(E)** Cumulative distribution of the inter-event intervals and the mean values of the frequency of mIPSCs were shown in the graph. **(F)** Cumulative distributions and mean values of the amplitude of mIPSCs were shown in the graph. **(G)** The scatter plot shows the relationship between the frequencies of mIPSC (x-axis) and mEPSC (y-axis). Each dot represents a single neuron from saline- or risperidone-injected SIK1-MT mice. **(H)** E/I balance index of saline (Sal)- and risperidone (Ris)-injected SIK1-MT mice is shown. E/I balance is unchanged between saline- and risperidone-injected SIK1-MT mice. **(I–K)** Resting membrane potential **(I)**, input resistance **(J)**, membrane capacitance **(K)** of pyramidal neurons in layer 5 of mPFC of saline (Sal)- and risperidone (Ris)-injected SIK1-MT mice. **(L–N)** half-peak width **(L)**, rise time **(M)**, and decay time **(N)** of the action potential of the pyramidal neurons in layer 5 of the mPFC of saline (Sal)- and risperidone (Ris)-injected SIK1-MT mice. **(O)** Representative traces of induced action potentials responded to 240 pA injected currents. **(P)** Graph for the relationship between spike frequency and injected current. Spike frequency of the action potential was decreased in risperidone-injected SIK1-MT mice. The numbers of neurons and mice used in each analysis are shown on the bar (neurons/mice) in the graphs. Statistical analysis was made by Student's *t*-test. Statistical significance was indicated by asterisks (*p < 0.05).

Next, to examine a drug used to ameliorate ASD symptoms, we focused on risperidone, which is the first FDA-approved medicine for some autistic symptoms to alleviate aggression and repetitive behaviors (Mcdougle et al., 2000, 2008; Gould et al., 2011; Peñagarikano et al., 2011). To date, it is still unclear what physiological effects are observed by the application of risperidone, and we first examined the pharmacological effects of risperidone on neurons on acute brain slices. Acute administration of risperidone significantly attenuated the frequency, but not amplitude, of mEPSCs in pyramidal neurons in layer 5 of the mPFC in SIK1-MT mice compared with the saline-injected condition (Figures 5A–C). But the excitatory and inhibitory (E/I) synaptic balance was unchanged because risperidone also reduced the frequency of mIPSCs in SIK1-MT mice (Figures 5D–H). The rise time and decay time constant of mEPSC and mIPSC were not altered by the application of risperidone (Supplementary Figure 6). We further examined the effect of risperidone on the excitability of pyramidal neurons in layer 5 of mPFC. Risperidone increased the membrane capacitance and decreased the frequency of action potential of the pyramidal neurons in layer 5 of the mPFC in SIK1-MT mice without altering resting potential, input resistance, and kinetics of action potential (Figures 5I–P).

### Risperidone Ameliorated Repetitive, but Not Social, Behavior in SIK1-MT Mice

**Figure 6:**
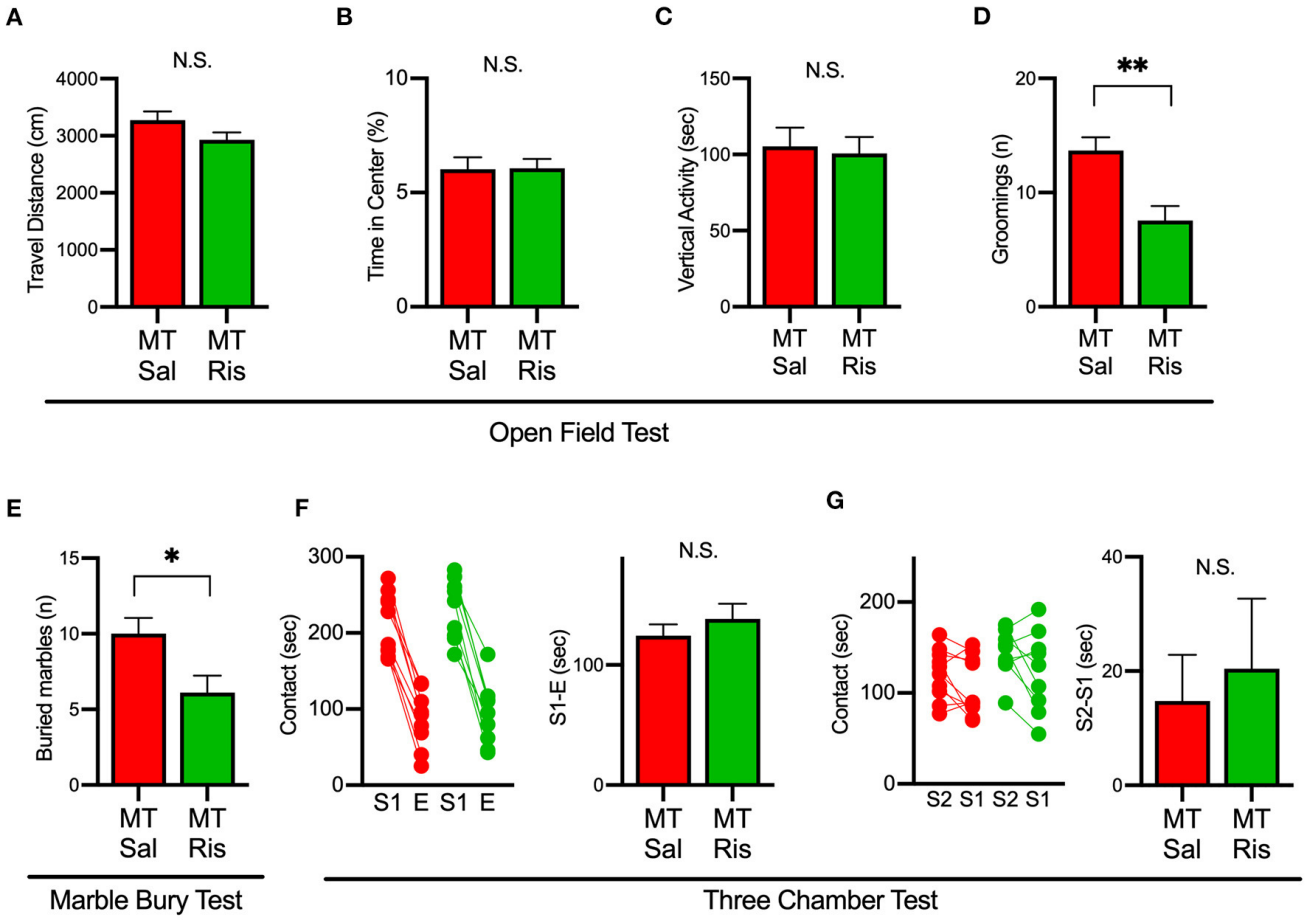
Administration of risperidone ameliorated increased repetitive behaviors but not social deficits in SIK1-MT mice. **(A)** Total travel distance of saline (Sal)- and risperidone (Ris)-injected SIK1-MT mice. **(B)** The percentage of the time spent in the center of the open field arena. **(C)** Total time of the vertical activities of saline (Sal, *n* = 10) and risperidone (Ris, *n* = 9) -injected SIK1-MT mice. **(D)** The number of groomings during the observation period. **(E)** The number of marbles buried in the marble burying test. The number of buried marbles was less in risperidone-injected SIK1-MT mice compared to that of saline-injected. **(F,G)** Sociability and the social novelty test between saline- and risperidone-injected SIK1-MT mice. Risperidone did not affect social behavior in SIK1-MT mice. The numbers of neurons and mice used in each analysis are shown on the bar (neurons/mice) in the graphs. Statistical analysis was made by Student's *t*-test. Statistical significance was indicated by asterisks (*p < 0.05 and **p < 0.01).

We next examined the effect of risperidone on the behavioral deficits observed in SIK1-MT mice. In the open field test, risperidone reduced the number of grooming in SIK1-MT mice, without affecting the travel distance, the time spent in the center, and the vertical activity (Figures 6A–D). The number of buried marble in the marble burying test was also reduced by risperidone treatment in SIK1-MT mice (Figure 6E). However, risperidone did not show any changes in social interactions observed in the three-chamber test compared with the saline treatment (Figures 6F,G).

## Discussion

SIK1 C-terminal-truncated mutations were identified in patients with EIEE-30 (Hansen et al., 2015). To study the effect of these mutations on the etiology of EIEE-30, we generated C-terminal-truncated SIK1-MT mice using CRISPR/Cas9-mediated genome editing as disease models. We studied these mice by focusing on the synaptic function and behaviors and found the following: (1) The frequency of mEPSCs and the neuronal excitability were increased in pyramidal neurons in layer 5 of the mPFC of SIK1-MT mice. (2) Repetitive behavior was increased and the social behavior was impaired in the SIK1-MT mice. (3) Elevated excitatory synaptic transmission and neural excitability in the SIK1-MT mice were restored by risperidone treatment. (4) Increased repetitive behavior, but not social behaviors, in SIK1-MT mice was ameliorated by risperidone treatment.

The mechanism by which the synaptic function of the SIK1-MT mice shifts to excitatory dominant remains unknown. SIK has been shown to regulate the subcellular localization and the biological activities of the class IIa histone deacetylases (HDAC4/5/7/9) (Berdeaux et al., 2007; Abend et al., 2017; Hsu et al., 2020) and CRTCs 1, 2, and 3 (Koo et al., 2005; Jagannath et al., 2013; Nixon et al., 2016) through the phosphorylation. SIK1-WT was distributed in the nucleus in a punctate pattern, whereas the SIK1-MT was diffused to the cytoplasm (Figure 1B). The altered subcellular distribution of SIK1 mutants may affect the expressions of genes related to ion channels or synaptic proteins (Pröschel et al., 2017), resulting in the increased excitability and excitatory synaptic inputs of neurons in SIK1-MT mice.

Application of risperidone attenuated the enhanced neural excitability and excitatory synaptic function in mPFC of SIK1-MT mice. We also found that the risperidone rescued increased repetitive behavior. The neuronal activity in mPFC has been linked to the repetitive behavior in rodent models (Takahata and Moghaddam, 2003; Aliane et al., 2009; Kim et al., 2016), which is supported by our findings in this study. Risperidone is the first FDA-approved drug for ASD used for ameliorating repetitive and aggressive symptoms of the disorder (Mcdougle et al., 2000, 2008). It antagonizes serotonin (5-HT2A, 5-HT1B, and 5-HT7) and dopamine D2 receptors (Nasrallah, 2008). Antipsychotic drugs, including risperidone, have been shown to attenuate voltage-gated sodium channel function, resulting in the reduction of activity-dependent synaptic vesicle release from presynaptic terminals (Tischbirek et al., 2012). The reduction of neural excitability observed in this study is consistent with the results. The reduction of spontaneous synaptic vesicle release by risperidone in this study may suggest the existence of other mechanisms by which risperidone affects the synaptic vesicle exocytosis directly or indirectly.

**Figure 7:**
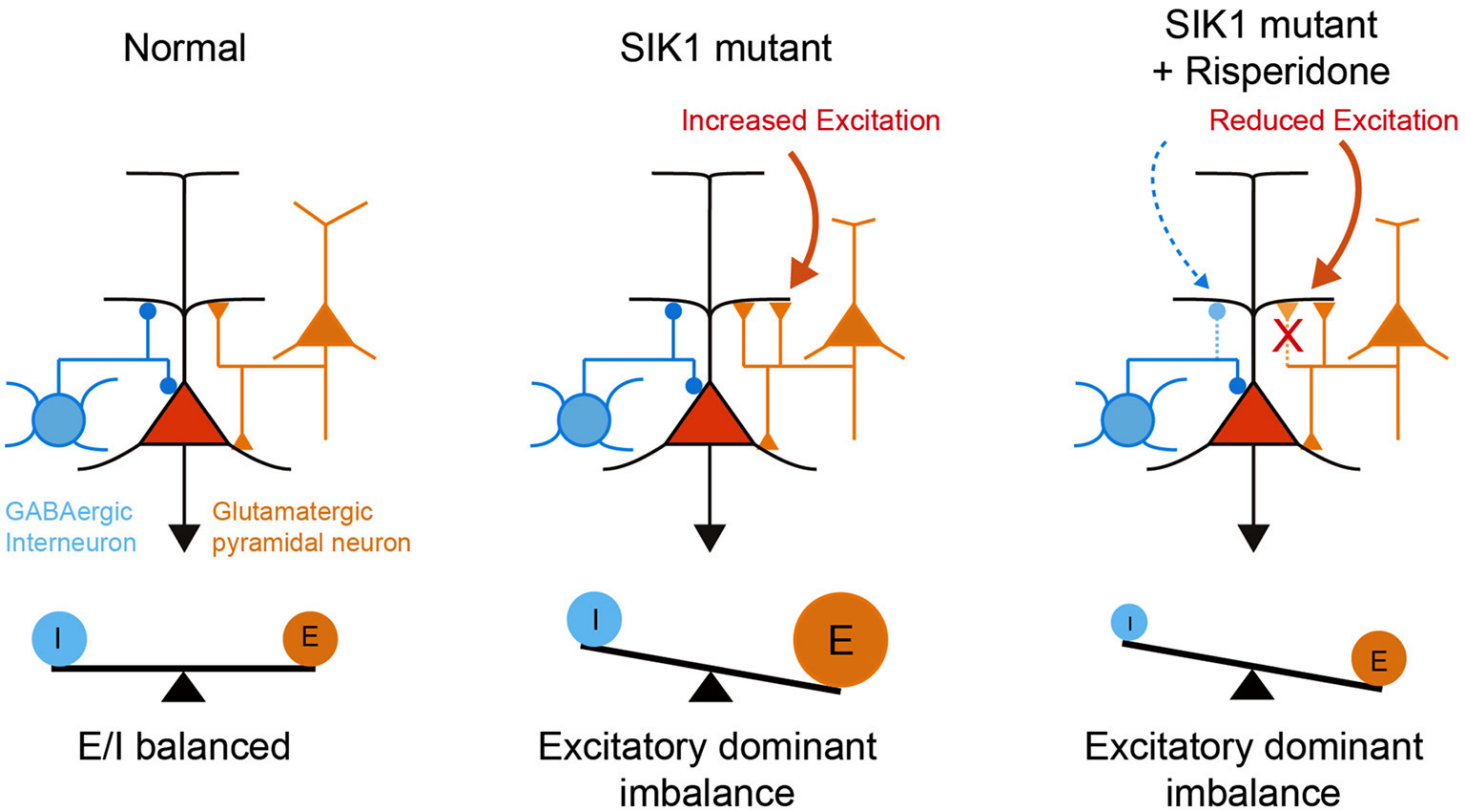
A schematic illustration of neuronal abnormality in SIK1-MT mice. In SIK1-MT mice, excitatory synaptic input onto pyramidal neurons in layer 5 of mPFC is increased, resulting in the disruption of E/I balance (middle). Risperidone restores the increased excitatory synaptic input. Risperidone also decreases the inhibitory input onto the pyramidal neurons. As a result, disrupted E/I balance is maintained in the risperidone-treated SIK1-MT mice (right).

Risperidone did not rescue deficits in social behavior in SIK1-MT mice, which is commonly observed in other animal models of neurodevelopmental disorders (Chadman, 2011; Gould et al., 2011; Peñagarikano et al., 2011; Bonini et al., 2016; Teng et al., 2016). It has been shown that the social behavioral deficit is attributable to the disruption of E/I synaptic balance in various animal models (Rubenstein and Merzenich, 2003; Tabuchi et al., 2007; Lee et al., 2017; Sohal and Rubenstein, 2019). We observed that risperidone also attenuated inhibitory synaptic function as well as excitatory synaptic function in mPFC, retaining disrupted E/I synaptic balance in SIK1-MT mice. The disrupted E/I synaptic balance after the administration of risperidone may be a possible mechanism that underlies the social behavioral deficits in SIK1-MT mice (Figure 7).

In summary, we generated and analyzed C-terminal-truncated SIK1-mutant mice (SIK1-MT) as models for EIEE-30. SIK1-MT mice exhibited social deficits and repetitive behavior consistent with the autistic phenotypes of EIEE-30. Elevated excitability of neurons and excitatory synaptic function were observed in the mPFC of SIK1-MT mice. Acute treatment with risperidone selectively restored the repetitive behavior, but not social deficits, likely due to the attenuation of elevated excitatory synaptic function. Reduction of the inhibitory synaptic function occurred by risperidone treatment, causing further E/I imbalance. Future studies that selectively attenuate excitatory synaptic function by optogenetics will be required to achieve the more efficient rescue of ASD symptoms, including social behavioral deficits in this model.

## Data Availability Statement

The original contributions generated for the study are included in the article/Supplementary Material, further inquiries can be directed to the corresponding author/s.

## Ethics Statement

The animal study was reviewed and approved by the Committee for Animal Experiments of Shinshu University.

## Author Contributions

TM, TYa, and KT designed experiments. TM and TYo generated SIK1-MT mice. MB and TM performed electrophysiology. TK, TM, and KN performed behavioral experiments. MB, TM, EK-S, and YS performed molecular experiments. TM and KT wrote manuscripts. All authors contributed to the article and approved the submitted version.

## Conflict of Interest

The authors declare that the research was conducted in the absence of any commercial or financial relationships that could be construed as a potential conflict of interest.
