# Supplementary material for: Risperidone Mitigates Enhanced Excitatory Neuronal Function and Repetitive Behavior Caused by an ASD-Associated Mutation of SIK1
Source: Front Mol Neurosci. 2021 Jul 6;14:706494. doi: 10.3389/fnmol.2021.706494 (PMC8289890; doi:10.3389/fnmol.2021.706494)
Supplement: Supplementary file 1 [file Data_Sheet_1.PDF]

List of Oligos

Primer #	Oligo name	sequence (5' to 3')	Purpose
1	SIK1-gRNA sense	ACCGGGGGAGTTCGGACGGAGGACG	pCGSapI-SIK1
	SIK1-gRNA anti	AAACGTCCTCCGTCCGAACTCCCCC	
2	IV-T3 Cas9 sense	CCAAGCTCGAAATTAACCCTCACTAAAGGGGGGGAGTTCGGACGGAGGAC	guide RNA for SIK1
	IV-T3 Cas9 anti	AAAAAAAGCACCGACTCGGTGCCACTTTTTCAAGT	
3	Geno-SIK1-sense	CCACATGGCAGGACACATCT	Genotyping
	Geno-SIK1-anti	TAAACCCCTGCCTGCTCTTG	Genotyping and Sanger sequencing
4	SIK1 cDNA sense	TCTAGACCATGGTGATCATGTCGGAGTTCAG	Subcloning of SIK1 cDNA
	SIK1 cDNA anti	GTTTAAACTCACTGTACCAGGACGAATGTCC	
5	mt-SIK1 sense	GTGTGCCGTCCGAACTCCCCGGG	Mutagenesis to produce SIK1-MT
	mt-SIK1 anti	GAGTTCGGACGGCACACCTGGCGAGCC	
6	Venus sense	ACATGATCACCATGGTGGCGTACTTGTACAGCTCG	Construction of Venus-SIK1
	Venus anti	GGCGAATTGGGCCCTCTAGACCATGGTGAGCAAGGG	
7	RT-PCR-SIK1 sense	CCTCCCCTTCTCTGCAAGTG	RT-PCR and Sanger sequencing
	RT-PCR-SIK1 anti	TGGTGCTGTAACTGGAGCAG	RT-PCR
