# Supplementary material for: Risperidone Mitigates Enhanced Excitatory Neuronal Function and Repetitive Behavior Caused by an ASD-Associated Mutation of SIK1
Source: Front Mol Neurosci. 2021 Jul 6;14:706494. doi: 10.3389/fnmol.2021.706494 (PMC8289890; doi:10.3389/fnmol.2021.706494)
Supplement: Supplementary file 2 [file Data_Sheet_2.PDF]

Supplementary Table 2

Figure	Parameter	Sample	Mean ± SE	Statistical Test
1C	Expression levels of mRNA	Brain sample WT	0.0173 ± 0.0008	ANOVA Bonferroni posthoc P < 0.0001 (vs hetero MT)
		Brain sample hetero MT	0.9466 ± 0.0117	
		Plasmid sample WT:MT = 1:1	0.9982 ± 0.0128	ANOVA Bonferroni posthoc P = 0.0835 (vs hetero MT)
		Plasmid sample WT:MT = 1:0.5	0.8111 ± 0.0141	ANOVA Bonferroni posthoc P < 0.0001 (vs hetero MT)
1D	Body weight at P3	WT male	2.21 ± 0.10 g	Student's t-test
		MT male	2.26 ± 0.09 g	P = 0.714
		WT female	2.27 ± 0.04 g	Student's t-test
		MT female	2.17 ± 0.10 g	P = 0.376
	Body weight at P5	WT male	3.03 ± 0.16 g	Student's t-test
		MT male	3.11 ± 0.10 g	P = 0.678
		WT female	3.25 ± 0.05 g	Student's t-test
		MT female	3.00 ± 0.14 g	P = 0.109
	Body weight at P7	WT male	4.04 ± 0.19 g	Student's t-test
		MT male	4.11 ± 0.14 g	P = 0.770
		WT female	4.32 ± 0.10 g	Student's t-test
		MT female	3.99 ± 0.21 g	P = 0.164
	Body weight at P9	WT male	4.85 ± 0.15 g	Student's t-test
		MT male	5.06 ± 0.11 g	P = 0.303
		WT female	4.95 ± 0.11 g	Student's t-test
		MT female	4.72 ± 0.17 g	P = 0.283
	Body weight at P11	WT male	5.91 ± 0.17 g	Student's t-test
		MT male	6.00 ± 0.13 g	P = 0.682
		WT female	5.99 ± 0.05 g	Student's t-test
		MT female	5.67 ± 0.21 g	P = 0.150
	Body weight at P13	WT male	6.68 ± 0.22 g	Student's t-test
		MT male	6.74 ± 0.15 g	P = 0.806
		WT female	6.48 ± 0.15 g	Student's t-test
		MT female	6.18 ± 0.27 g	P = 0.358
	Body weight at P15	WT male	6.95 ± 0.24 g	Student's t-test
		MT male	7.11 ± 0.16 g	P = 0.582
		WT female	6.52 ± 0.29 g	Student's t-test
		MT female	6.42 ± 0.36 g	P = 0.822
1G	Frequency of mEPSC	WT	0.57 ± 0.08 Hz	Student's t-test
		MT	1.28 ± 0.12 Hz	P < 0.0001
1H	Amplitude of mEPSC	WT	9.44 ± 0.37 pA	Student's t-test
		MT	9.31 ± 0.40 pA	P = 0.813
1J	Frequency of mIPSC	WT	1.29 ± 0.15 Hz	Student's t-test
		MT	1.42 ± 0.12 Hz	P = 0.500
1K	Amplitude of mIPSC	WT	14.93 ± 0.73 pA	Student's t-test
		MT	13.88 ± 0.54 pA	P = 0.262
1M	E/I balance index	WT	0.316 ± 0.054	Student's t-test
		MT	0.465 ± 0.034	P = 0.029
S3B	Rise time of mEPSC	WT	1.770 ± 0.062 ms	Student's t-test
		MT	1.580 ± 0.0.069 ms	P = 0.901
S3C	Decay time of mEPSC	WT	0.920 ± 0.158 ms	Student's t-test
		MT	1.750 ± 0.143 ms	P = 0.241
S3E	Rise time of mIPSC	WT	2.550 ± 0.064 ms	Student's t-test
		MT	2.260 ± 0.086 ms	P = 0.570
S3F	Decay time of mIPSC	WT	5.330 ± 0.901 ms	Student's t-test
		MT	5.180 ± 0.810 ms	P = 0.960
	excitatory PPR 30 ms-interval	WT	1.255 ± 0.086	Student's t-test
		MT	1.154 ± 0.097	P = 0.445
	excitatory PPR	WT	1.173 ± 0.060	Student's t-test

S4B	50 ms-interval	MT	1.221 ± 0.143	P = 0.751
	excitatory PPR 100 ms-interval	WT	1.073 ± 0.056	Student's t-test
		MT	1.162 ± 0.099	P = 0.431
	excitatory PPR 200 ms-interval	WT	0.946 ± 0.053	Student's t-test
		MT	1.131 ± 0.086	P = 0.076
S4D	inhibitory PPR 30 ms-interval	WT	0.534 ± 0.031	Student's t-test
		MT	0.454 ± 0.036	P = 0.104
	inhibitory PPR 50 ms-interval	WT	0.624 ± 0.025	Student's t-test
		MT	0.553 ± 0.030	P = 0.077
	inhibitory PPR 100 ms-interval	WT	0.763 ± 0.021	Student's t-test
		MT	0.736 ± 0.028	P = 0.436
	inhibitory PPR 200 ms-interval	WT	0.817 ± 0.016	Student's t-test
		MT	0.809 ± 0.042	P = 0.850
S5	NMDA/AMPA ratio	WT	1.482 ± 0.119	Student's t-test
		MT	1.442 ± 0.130	P = 0.823
2A	Resting membrane potential	WT	-67.19 ± 1.22 mV	Student's t-test
		MT	-66.26 ± 1.19 mV	P = 0.590
2B	Input Resistance	WT	122.3 ± 7.04 mΩ	Student's t-test
		MT	152.9 ± 6.85 mΩ	P = 0.0047
2C	Membrane capacitance	WT	54.80 ± 6.99 pF	Student's t-test
		MT	31.70 ± 2.45 pF	P = 0.0047
2D	Threshold of action potential	WT	-35.41 ± 1.84 mV	Student's t-test
		MT	-31.38 ± 1.76 mV	P = 0.126
2E	Half peak width of action potential	WT	3.208 ± 0.152 ms	Student's t-test
		MT	3.200 ± 0.148 ms	P = 0.971
2F	Rise time of action potential	WT	1.500 ± 0.034 ms	Student's t-test
		MT	1.485 ± 0.027 ms	P = 0.727
2G	Decay time of action potential	WT	12.95 ± 1.544 ms	Student's t-test
		MT	9.569 ± 0.234 ms	P = 0.040
2I	Spike frequency (150 pA)	WT	3.969 ± 1.080 Hz	Student's t-test
		MT	6.454 ± 1.340 Hz	P = 0.162
	Spike frequency (180 pA)	WT	8.385 ± 1.251 Hz	Student's t-test
		MT	11.91 ± 1.060 Hz	P = 0.042
	Spike frequency (210 pA)	WT	12.67 ± 1.048 Hz	Student's t-test
		MT	16.96 ± 1.313 Hz	P = 0.017
	Spike frequency (240 pA)	WT	14.88 ± 0.906 Hz	Student's t-test
		MT	22.93 ± 3.620 Hz	P = 0.041
3B	Travel distance	WT	3,206 ± 154.9 cm	Student's t-test
		MT	3,257 ± 211.3 cm	P = 0.844
3C	Time spent in the center	WT	6.456 ± 0.556 %	Student's t-test
		MT	4.680 ± 0.664 %	P = 0.058
3D	Vertical activity	WT	90.22 ± 5.64 sec	Student's t-test
		MT	79.71 ± 6.83 sec	P = 0.251
3E	Groomings	WT	7.556 ± 0.973	Student's t-test
		MT	16.86 ± 3.011	P = 0.006
3G	Entry to open arm	WT	13.67 ± 1.054	Student's t-test
		MT	15.71 ± 0.918	P = 0.179
3I	Buried marbles	WT	3.667 ± 0.799	Student's t-test
		MT	11.29 ± 1.658	P = 0.0005
3K	Socialbility WT's contact	S1	231.9 ± 6.90 sec	Student's t-test
		E	76.56 ± 9.29 sec	P < 0.0001
	Socialbility MT's contact	S1	205.7 ± 11.07 sec	Student's t-test
		E	88.10 ± 6.56 sec	P < 0.0001
	Socialbility S1-E	WT	155.3 ± 14.36 sec	Student's t-test
		MT	117.6 ± 14.23 sec	P = 0.080
3M	Social Novelty WT's contact	S2	166.4 ± 15.22 sec	Student's t-test
		S1	93.22 ± 5.20 sec	P = 0.0046
	Social Novelty MT's contact	S2	113.6 ± 7.20 sec	Student's t-test
		S1	105.7 ± 9.47 sec	P = 0.571
	Social Novelty S2-S1	WT	73.22 ± 18.84 sec	Student's t-test
		MT	7.90 ± 13.44 sec	P = 0.011

S6C	USV at P5	WT	229.9 ±62.00	Student's t-test
		MT	150.1 ± 49.03	P = 0.341
	USV at P8	WT	180.6 ± 55.07	Student's t-test
		MT	161.7 ± 65.96	P = 0.828
	USV at P11	WT	299.6 ± 49.64	Student's t-test
		MT	241.7 ± 89.02	P = 0.567
	USV at P14	WT	467.1 ± 122.5	Student's t-test
		MT	410.7 ± 113.9	P = 0.744
4B	Frequency of mEPSC	MT-Sal	1.134 ± 0.157 Hz	Student's t-test
		MT-Ris	0.689 ± 0.106 Hz	P = 0.027
4C	Amplitude of mEPSC	MT-Sal	9.503 ± 0.462 pA	Student's t-test
		MT-Ris	8.708 ± 0.330 pA	P = 0.171
4E	Frequency of mIPSC	MT-Sal	1.940 ± 0.364 Hz	Student's t-test
		MT-Ris	1.092 ± 0.266 Hz	P = 0.072
4F	Amplitude of mIPSC	MT-Sal	13.32 ± 0.863 pA	Student's t-test
		MT-Ris	12.90 ± 0.710 pA	P = 0.712
4H	E/I balance index	MT-Sal	0.3676 ± 0.042	Student's t-test
		MT-Ris	0.4438 ± 0.051	P = 0.277
4I	Resting membrane potential	MT-Sal	-71.73 ± 2.32 mV	Student's t-test
		MT-Ris	-67.68 ± 2.63 mV	P = 0.259
4J	Input Resistance	MT-Sal	164.5 ± 17.1 mΩ	Student's t-test
		MT-Ris	151.1 ± 12.6 mΩ	P = 0.534
4K	Membrane capacitance	MT-Sal	27.18 ± 4.39 pF	Student's t-test
		MT-Ris	46.73 ± 7.35 pF	P = 0.032
4L	Half peak width of action potential	MT-Sal	2.817 ± 0.158 ms	Student's t-test
		MT-Ris	3.333 ± 0.220 ms	P = 0.069
4M	Rise time of action potential	MT-Sal	1.475 ± 0.030 ms	Student's t-test
		MT-Ris	1.433 ± 0.022 ms	P = 0.283
4N	Decay time of action potential	MT-Sal	7.925 ± 0.437 ms	Student's t-test
		MT-Ris	8.092 ± 0.585 ms	P = 0.822
4P	Spike frequency (150 pA)	MT-Sal	8.45 ± 1.310 Hz	Student's t-test
		MT-Ris	5.40 ± 1.465 Hz	P = 0.137
	Spike frequency (180 pA)	MT-Sal	12.65 ± 0.809 Hz	Student's t-test
		MT-Ris	9.56 ± 1.395 Hz	P = 0.074
	Spike frequency (210 pA)	MT-Sal	15.73 ± 0.792 Hz	Student's t-test
		MT-Ris	13.38 ± 1.033 Hz	P = 0.060
	Spike frequency (240 pA)	MT-Sal	18.33 ± 0.769 Hz	Student's t-test
		MT-Ris	15.51 ± 0.990 Hz	P = 0.037
S7B	Rise time of mEPSC	MT-Sal	2.085 ± 0.065 ms	Student's t-test
		MT-Ris	2.005 ± 0.072 ms	P = 0.424
S7C	Decay time of mEPSC	MT-Sal	1.834 ± 0.133 ms	Student's t-test
		MT-Ris	2.040 ± 0.197 ms	P = 0.403
S7E	Rise time of mIPSC	MT-Sal	2.497 ± 0.047 ms	Student's t-test
		MT-Ris	2.585 ± 0.043 ms	P = 0.184
S7F	Decay time of mIPSC	MT-Sal	7.959 ± 0.558 ms	Student's t-test
		MT-Ris	9.479 ± 0.793 ms	P = 0.141
5A	Travel distance	MT-Sal	3,272 ± 156.9 cm	Student's t-test
		MT-Ris	2,927 ± 132.0 cm	P = 0.115
5B	Time spent in the center	MT-Sal	6.03 ± 0.521 %	Student's t-test
		MT-Ris	6.07 ± 0.412 %	P = 0.959
5C	Vertical activity	MT-Sal	105.5 ± 12.13 sec	Student's t-test
		MT-Ris	100.8 ± 10.75 sec	P = 0.776
5D	Grooming	MT-Sal	13.70 ± 1.155	Student's t-test
		MT-Ris	7.556 ± 1.281	P = 0.0023
5E	Buried marbles	MT-Sal	10.00 ± 1.054	Student's t-test
		MT-Ris	6.111 ± 1.124	P = 0.0218
5F	Socialbility MT-Sal's contact	S1	211.6 ± 12.79 sec	Student's t-test
		E	87.10 ± 11.29 sec	P < 0.0001
	Socialbility MT-Ris's contact	S1	231.6 ± 13.34 sec	Student's t-test
		E	93.11 ± 13.61 sec	P < 0.0001
	Socialbility	MT-Sal	124.5 ± 9.33 sec	Student's t-test

	S1-E	MT-Ris	138.4 ± 12.61 sec	P = 0.380
5G	Social Novelty<br>WT-Sal's contact	S2	121.1 ± 8.78 sec	Student's t-test<br>P = 0.099
		S1	106.3 ± 10.09 sec	
	Social Novelty<br>MT-Ris's contact	S2	144.4 ± 8.59 sec	Student's t-test<br>P = 0.133
		S1	124.0 ± 14.76 sec	
	Social Novelty<br>S2-S1	MT-Sal	14.80 ± 8.04 sec	Student's t-test<br>P = 0.699
		MT-Ris	20.44 ± 12.23 sec	
