# Supplementary figures and images for: Risperidone Mitigates Enhanced Excitatory Neuronal Function and Repetitive Behavior Caused by an ASD-Associated Mutation of SIK1
Source: Front Mol Neurosci. 2021 Jul 6;14:706494. doi: 10.3389/fnmol.2021.706494 (PMC8289890; doi:10.3389/fnmol.2021.706494)

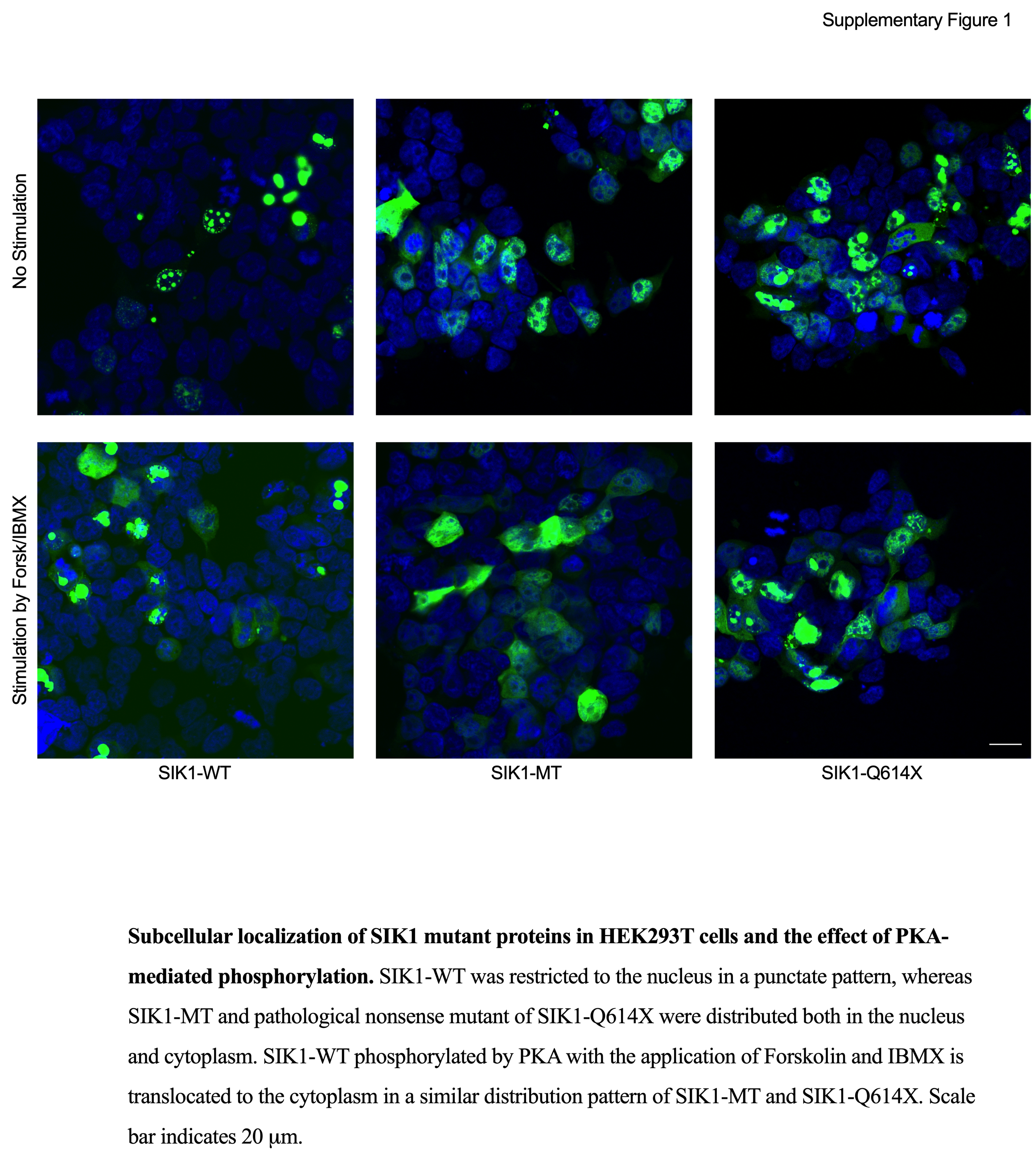

Supplement: Supplementary file 3 [file Image_1.TIFF]

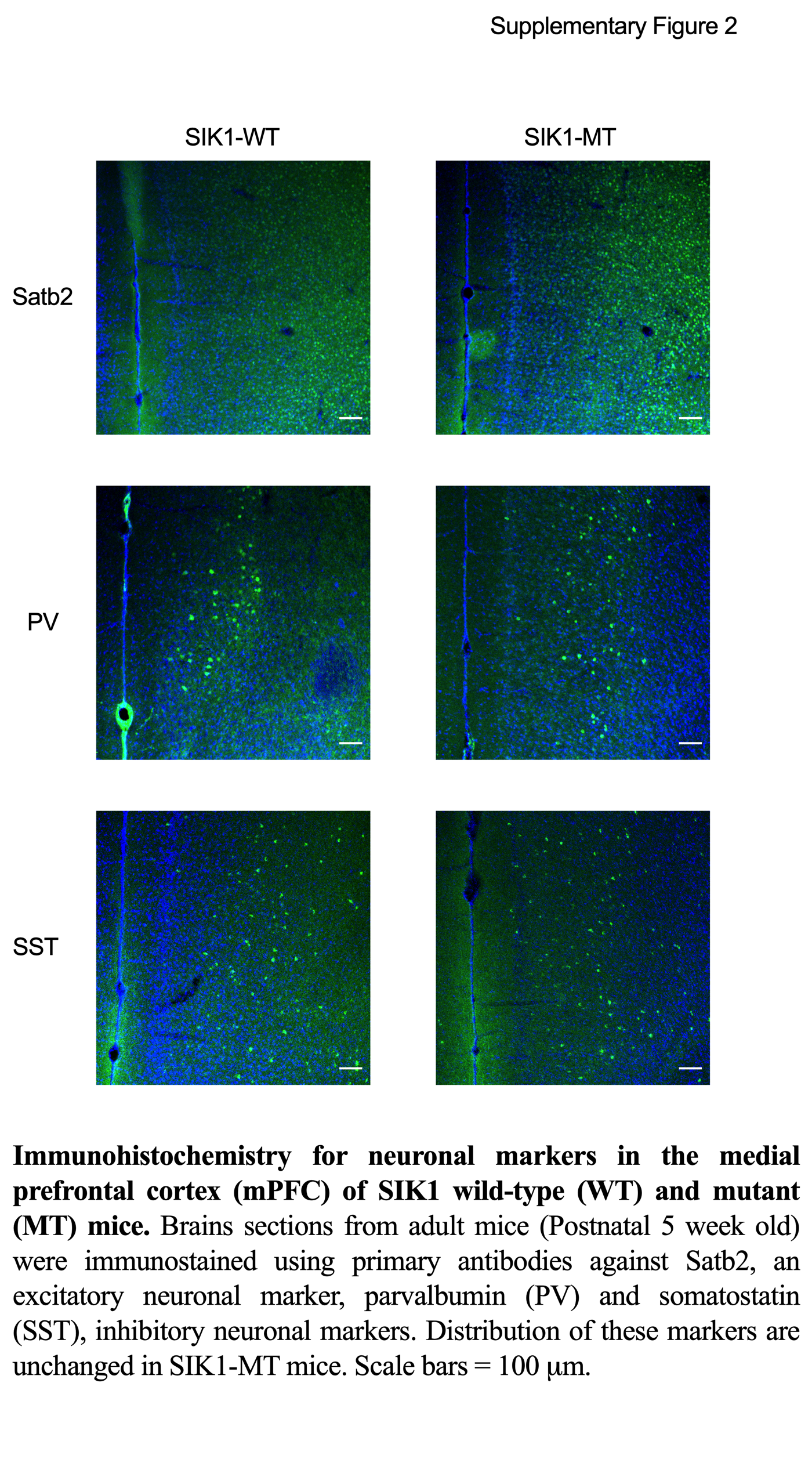

Supplement: Supplementary file 4 [file Image_2.TIFF]

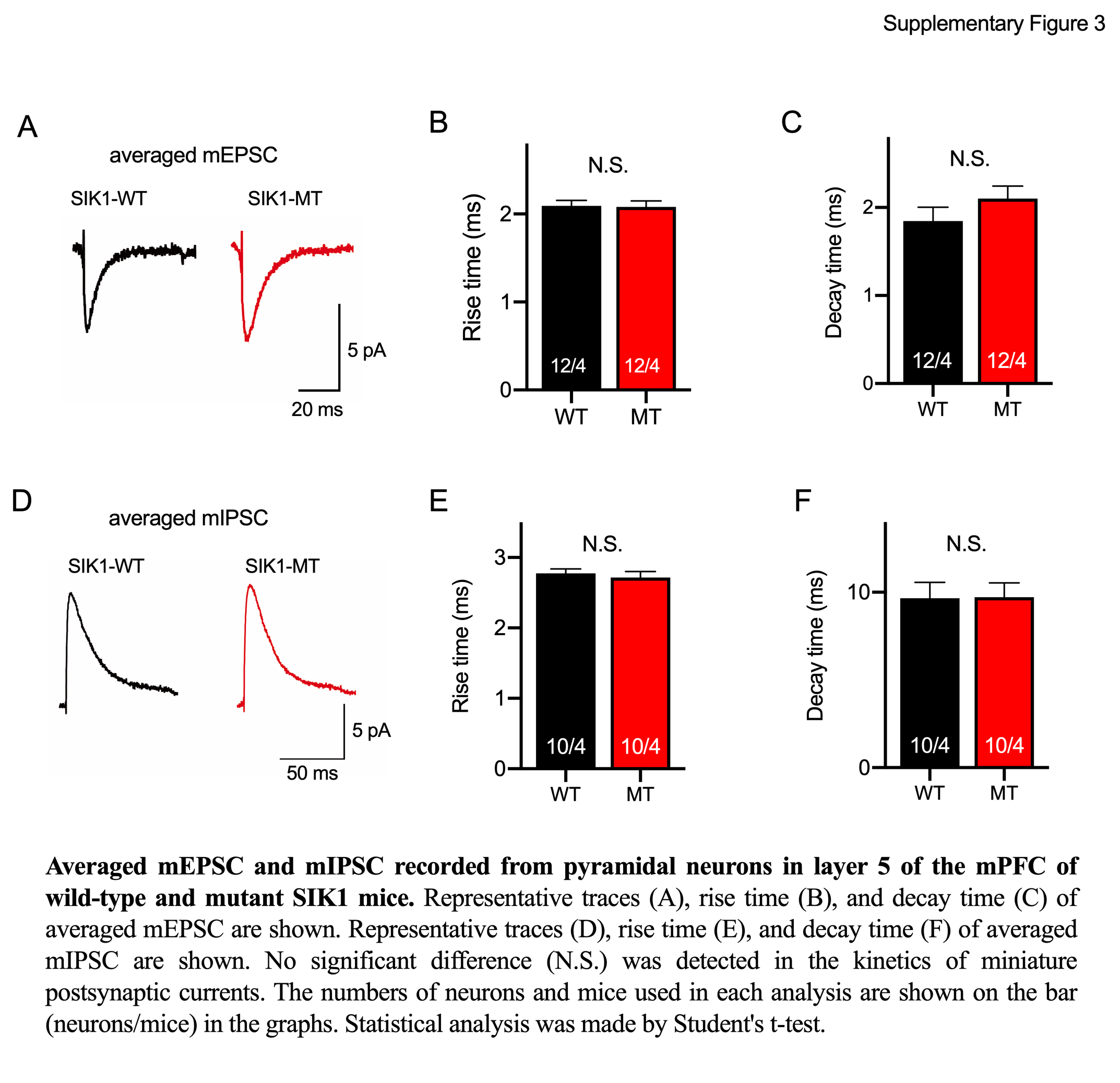

Supplement: Supplementary file 5 [file Image_3.TIFF]

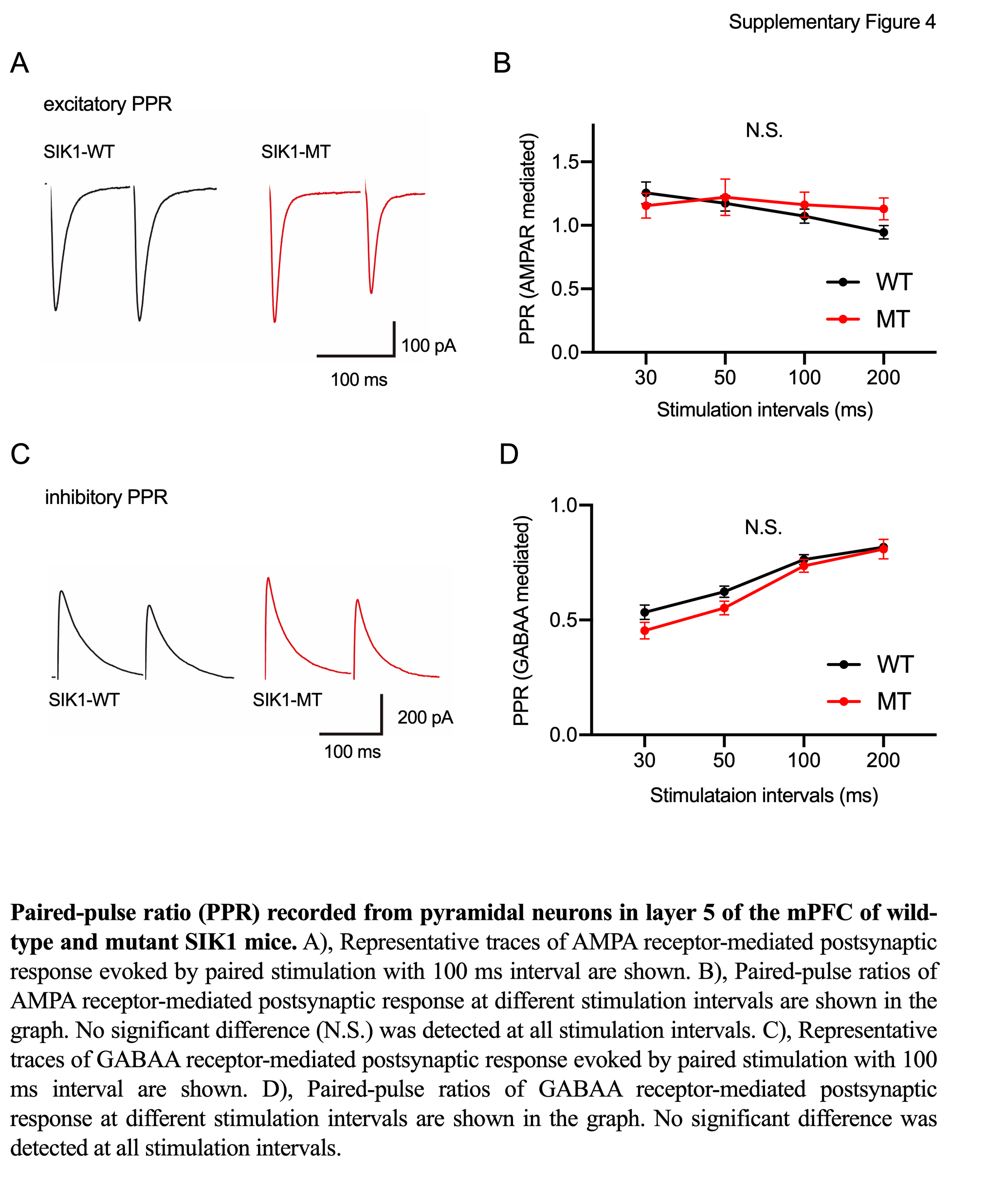

Supplement: Supplementary file 6 [file Image_4.TIFF]

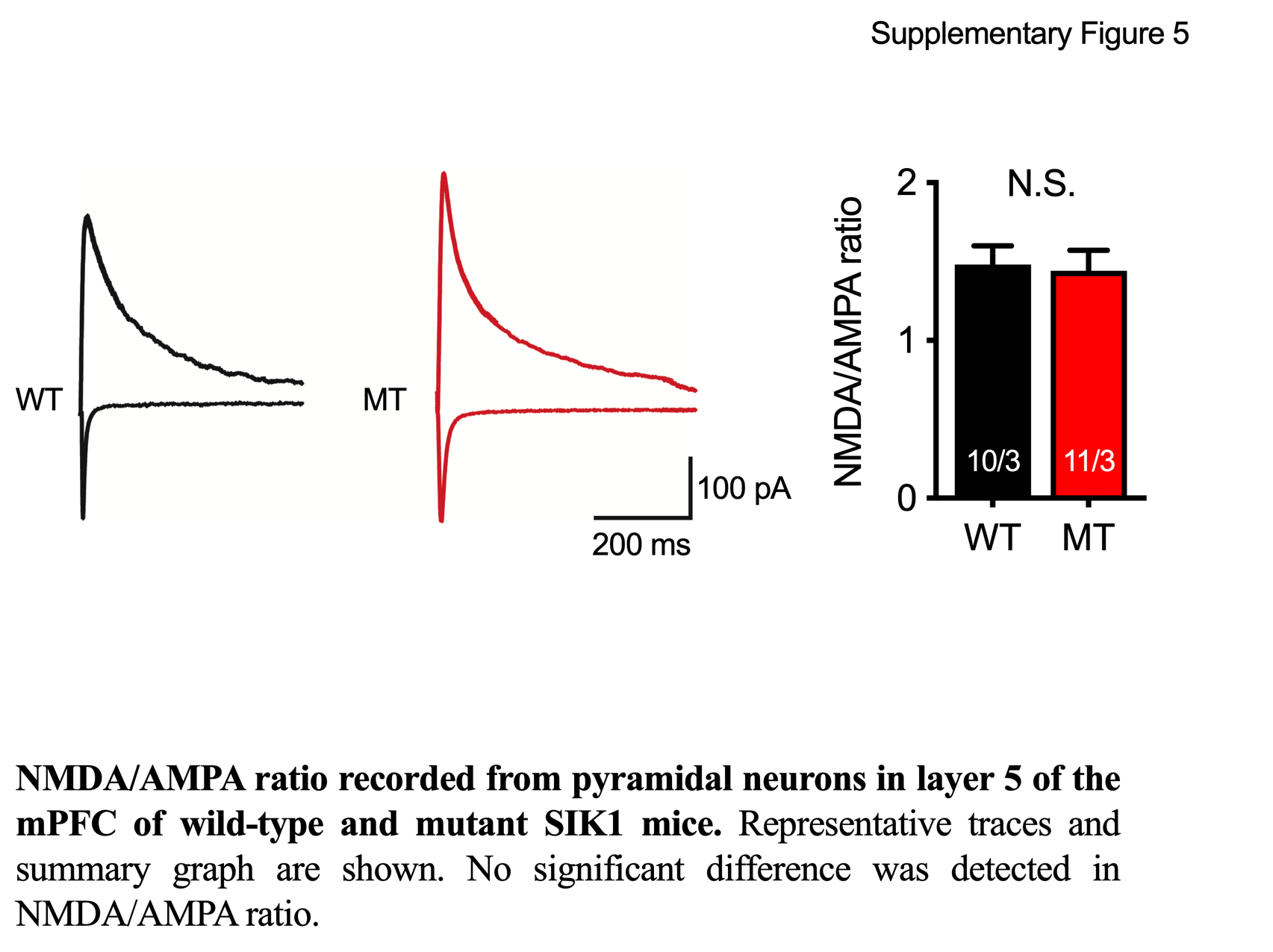

Supplement: Supplementary file 7 [file Image_5.TIFF]

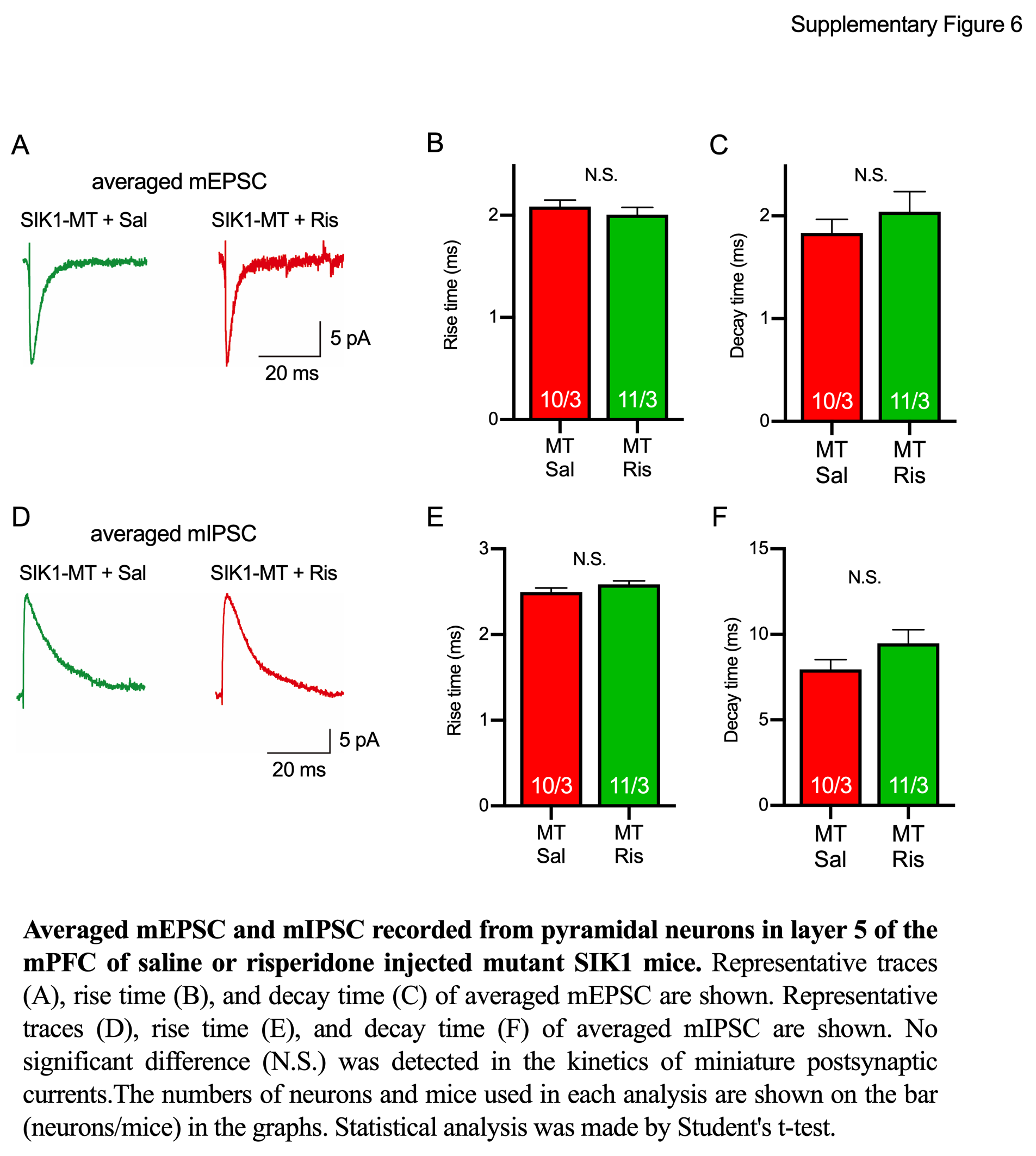

Supplement: Supplementary file 8 [file Image_6.TIFF]
